# Exploring the Effect and Mechanism of Liraglutide in Treating Depression Based on Network Pharmacology and Experimental Analysis

**DOI:** 10.1111/jcmm.70630

**Published:** 2025-06-03

**Authors:** Jiangjin Sun, Xiying Fu, Yaqi Liu, Tian Wang, Xing Zhao, Ranji Cui, Wei Yang

**Affiliations:** ^1^ Jilin Provincial Key Laboratory on Molecular and Chemical Genetic The Second Clinical Medical College of Jilin University (The Second hospital of Jilin University) Changchun China; ^2^ Department of Neurology The Second Clinical Medical College of Jilin University (The Second hospital of Jilin University) Changchun China; ^3^ Department of Endocrinology The Second Clinical Medical College of Jilin University (The Second Hospital of Jilin University) Changchun China

**Keywords:** CUMS, depression, liraglutide, microglia, network pharmacology, neuroinflammation

## Abstract

Depression is a disorder caused by various reasons, with low mood as the main symptom, and it has a serious impact on mental health. Liraglutide (Lir) has been confirmed to alleviate neuroinflammation and depression‐like behaviours induced by chronic stress, but its underlying mechanisms remain unclear. This study investigated the regulation of Lir for microglia‐associated inflammation in depression through network pharmacology. In vivo experiments demonstrate that Lir reduces depressive‐like behaviours by activating Nrf2 and subsequently downregulating HMGB1 expression, while also reducing the generation of pro‐inflammatory mediators and oxidative stress damage. In vitro studies confirmed that the downregulation of HMGB1 depends on Nrf2 activation, and Lir activates Nrf2 via the PI3K/AKT pathway. Additionally, indirect co‐culture of BV2 and HT22 cells demonstrated Lir's neuroprotective effects against neuronal apoptosis, consistent with findings from in vivo experiments. The study results first demonstrate that Lir exerts antidepressant effects through the PI3K/Nrf2/HMGB1 pathway, which reveals a novel mechanism of action for the antidepressant effects of Lir.

## 1 Introduction

The mechanisms of depression are complex, and scholars have increasingly focused on the connection between neuroinflammation and depression [1]. As the primary phagocytes of the brain, microglia can secrete cytokines, chemokines and growth factors in response to stressors under physiological conditions to maintain the stability of the brain microenvironment [2, 3]. However, under pathological conditions, excessively activated microglia will secrete large amounts of inflammatory cytokines, leading to neuroinflammation and eventually severe neuronal damage [4].

Animal studies have shown that depressive‐like behaviours in rodents are closely associated with microglial activation [5]. When rats are impacted by chronic stress, they not only exhibit depressive‐like behaviours but also show a substantial activation of microglia in the hippocampus [6]. Furthermore, chronic stress‐induced depressive‐like behaviours in rodents are related to the activation state, number, morphology and function of microglia [7, 8]. Other studies have demonstrated that reducing pro‐inflammatory cytokine levels and increasing anti‐inflammatory cytokine levels can effectively suppress central neuroinflammation and reduce depressive‐like behaviours in mice [9, 10]. These findings indicate that microglial activation is a crucial mediator of central neuroinflammation, leading to the development of depression.

The glucagon‐like peptide‐1 (GLP‐1) primarily derived from intestinal L cells, a crucial incretin in the gut‐pancreatic axis, can be found in both peripheral tissues and various regions of the brain [11, 12]. GLP‐1 and its analogues can cross the blood–brain barrier and reduce levels of inflammatory factors in both brain tissue and the peripheral systems [13, 14, 15]. Studies have shown that the GLP‐1R‐related pathway expressed in microglia can reduce inflammation, promote neurogenesis, provide neurotrophic effects and restore insulin signalling in neurons [12, 16, 17, 18]. GLP‐1RAs ameliorate neurodegenerative diseases associated with microglia‐mediated inflammation [19]. Additionally, GLP‐1 can decrease lipopolysaccharide (LPS)‐induced microglial activation and reduce inflammatory factor levels [20, 21]. In summary, the relationship between GLP‐1 and microglia reveals its potential in treating depression associated with neuroinflammation.

We utilised network pharmacology to identify key targets such as NFE2L2 (also known as Nrf2) and HMGB1. Previous studies have shown that Nrf2, as a transcription factor, regulates redox homeostasis and inhibits processes, such as neuroinflammation, ferroptosis and mitochondrial dysfunction [22]. Dysregulation of the Nrf2 pathway may lead to depression; targeting Nrf2 holds significant potential for depression treatment, but the specific mechanisms remain to be explored [23]. HMGB1 is a highly conserved nuclear DNA‐binding protein that can induce and amplify non‐infectious inflammatory responses [24]. Increased levels of HMGB1 can increase the number of pro‐inflammatory microglia. During inflammation, HMGB1 can be actively secreted by inflammatory cells or released from damaged and necrotic cells into the extracellular environment, where it interacts with toll‐like receptors (TLRs) on microglia, induces immune responses and promotes the production of inflammatory cytokines [25, 26, 27]. Thus, inhibition of HMGB1 is expected to be one of the therapeutic approaches for depression associated with neuroinflammation.

This study explored the regulation of Lir and microglia‐associated inflammation in depression through network pharmacology and both in vivo and in vitro experiments. The findings demonstrated that Lir might influence neuroinflammation and oxidative stress associated with microglia via the PI3K/Nrf2/HMGB1 signalling pathway and provides a potential approach for the treatment of depression.

## 2 Materials and Methods

### 2.1 Network Pharmacology

The chemical structure of the Lir was searched from the PubChem open chemistry database. Target information was then obtained using the SEA database, Swiss Target Prediction and TargetNet. Standard gene names were converted using the Universal Protein Resource. Depression‐related genes were selected from databases, such as GeneCards, OMIM and CTD. The drug and disease targets were compared using the Venny diagram to identify potential targets for Lir in treating neuroinflammation‐related depression. These targets were further analysed through a Protein–Protein Interaction (PPI) network using STRING and Cytoscape 3.9.1 software. Finally, Gene Ontology (GO) and Kyoto Encyclopedia of Genes and Genomes (KEGG) pathway enrichment analyses were performed using the DAVID database. Bar and bubble charts were generated using the Bioinformatics platform. Detailed information can be found in the Supporting Information.

### 2.2 Experimental Animals and Drugs

Male C57BL/6 mice (8–10 weeks, 18–23 g) were purchased from Liaoning Changsheng Life Sciences Ltd. (Benxi, Liaoning, China) and housed under standard conditions (12‐h light–dark cycle, 22°C ± 2°C), with enough food and water. All experiments followed ethical guidelines and were approved by the Ethics Committee of Jilin University (approval no. 2020(150); December 9, 2020). Lir (Novo Nordisk, Denmark) and Fluoxetine (Flx, PHR1394, Sigma‐Aldrich, USA) were dissolved in physiological saline, whereas the Nrf2 inhibitor ML385 (HY‐100523; Med Chem Express, China), the HMGB1 inhibitor Glycyrrhizin (GL, 50531; Sigma‐Aldrich, USA) and the PI3K pathway inhibitor LY294002 (HY‐10108; Med Chem Express, China) were prepared as a stock solution in DMSO. All drugs were diluted to the correct concentrations before use.

### 2.3 Experimental Design

#### 2.3.1 Animal Experiments

##### 2.3.1.1 Experiment Groups and Drug Dose

In the first experiment, mice were randomly divided into six groups (*n* = 10 per group): Control, CUMS, CUMS + low‐dose Lir, CUMS + medium‐dose Lir, CUMS + high‐dose Lir and CUMS + fluoxetine.

In the second experiment, mice were randomly divided into four groups (*n* = 13 per group): Control, CUMS, CUMS + Lir and CUMS + Lir + ML385.

In the third experiment, mice were randomly divided into four groups (*n* = 10 per group): Control, CUMS, CUMS + Lir and CUMS + GL.

Detailed drug dosages and experimental procedures are shown in the Supporting Information.

##### 2.3.1.2 The Process of Chronic Unpredictable Mild Stress (CUMS)

Mice were subjected to one of the following stressors randomly each day for four consecutive weeks to establish the CUMS depression model [28, 29]: Cage tilt for 24 h, cold water swim for 3 min (0°C), food or water deprivation for 24 h, horizontal shaking for 15 min, tail pinch for 1 min (1 cm from the tip of the tail), hot water stress for 3 min (45°C), wet bedding for 24 h, light or dark stimulation for 24 h, exposure to a novel odour environment for 6 h, confinement in a 50 mL centrifuge tube for 2 h. Control group mice were not subjected to any stressors.

##### 2.3.1.3 Behavioural Tests

The total distance travelled and movement speed in the OFT were used as indicators to assess the spontaneous activity of mice, and the FST and TST were used to evaluate the depressive state. Details of the process can be found in the Supporting Information.

#### 2.3.2 In Vitro Experiments

##### 2.3.2.1 Process of Cell Culture

The culture process of BV2 and HT22 cell lines is detailed in the Supporting Information.

##### 2.3.2.2 Method of Cell Viability Assay

We assessed cell viability using the CCK8 assay kit, and the detailed procedure is provided in the Supporting Information.

##### 2.3.2.3 Indirect Co‐Culture of BV2 and HT22 Cells

The culture media from the BV2 cell groups (control, LPS, LPS + Lir) in 100 mm dishes were collected and centrifuged at 1000 rpm for 5 min. The supernatants were then added to HT22 cells that had reached 70% cell density. After 24 h of incubation, the HT22 cells were collected for further experiments.

#### 2.3.3 Western Blot (WB)

Hippocampal (Hip) and prefrontal cortex (PFC) tissue or cells were lysed in RIPA buffer containing 1% PMSF, then the samples were sonicated and centrifuged at 12,000 rpm for 20 min at 4°C. The supernatant was collected and mixed with loading buffer, and the mixture was denatured by heating at 95°C for 10 min. Nuclear and cytoplasmic proteins were extracted using a commercial kit. Protein samples were separated on 10% SDS‐polyacrylamide gels and transferred onto polyvinylidene fluoride membranes. The membranes were blocked with 5% nonfat milk for 1 h and then incubated overnight at 4°C with the following primary antibodies. The membranes were then washed three times with TBST for 5 min each and incubated with corresponding secondary antibodies at room temperature for 1 h. Protein bands were detected using enhanced chemiluminescence. The optical density of the protein bands was analysed using ImageJ software. The specific information about the antibodies is provided in the Supporting Information.

#### 2.3.4 Process for Paraffin Embedding and Sectioning of Brain Tissue

The process of brain tissue paraffin embedding and sectioning is described in the Supporting Information.

#### 2.3.5 Haematoxylin and Eosin (H&E) Staining and Nissl Staining

Brain slices were baked, deparaffinised and rehydrated, then stained with H&E and Nissl; the methods can be found in the Supporting Information.

#### 2.3.6 Immunofluorescent Staining

After behavioural tests, the mice were anaesthetised for perfusion. Then, the entire brain was carefully removed and immersed in 4% PFA, fixed at room temperature overnight. The brain was dehydrated using a sucrose gradient of 10%, 20% and 30%, embedded and stored at −80°C for use. The frozen mouse brain was then placed in a frozen slicer to cut 20 μm brain tissue sections for immunofluorescence staining. Detailed steps can be found in the Supporting Information.

#### 2.3.7 Method of Enzyme‐Linked Immunosorbent Assay (ELISA)

The levels of Interleukin‐1β (IL‐1β), Interleukin‐6 (IL‐6), Tumour Necrosis Factor‐α (TNF‐α) and peripheral serum corticosterone in mouse Hip and PFC tissues were measured using an ELISA kit. Detailed steps can be found in the Supporting Information.

#### 2.3.8 Detection of Oxidative Stress Factor Levels

The levels of oxidative stress factors MDA, GSH and SOD were measured according to the kit instructions. The detailed procedure is provided in the Supporting Information.

#### 2.3.9 Method of Quantitative Real‐Time PCR (qRT‐PCR)

The experimental method of qRT‐PCR is described in the Supporting Information. The primer sequences are listed in Table S1.

#### 2.3.10 Tyramide Signal Amplification (TSA) for Immunofluorescence Double Staining

After drug treatment of BV2 cells, TSA immunofluorescence double staining was performed. The detailed method is provided in the Supporting Information.

#### 2.3.11 Detection of Reactive Oxygen Species (ROS) Levels

BV2 cells were cultured in six‐well plates, and ROS levels were detected using a ROS kit. The experimental method is described in the Supporting Information.

### 2.4 Statistical Analysis

All data were analysed using SPSS 23.0 statistical software and plotted using GraphPad Prism 9.0. For normally distributed data, one‐way analysis of variance (ANOVA) was applied, followed by post hoc comparisons using Bonferroni or Tamhane's T2 test. Non‐normally distributed data were analysed using the Kruskal–Wallis test for inter‐group comparisons. Statistical significance was set at *p* < 0.05 (*p < 0.05, **p < 0.01, ***p < 0.001).

## 3 Results

### 3.1 Network Pharmacology

#### 3.1.1 The Potential Targets of Lir in the Treatment of Depression

**FIGURE 1:**
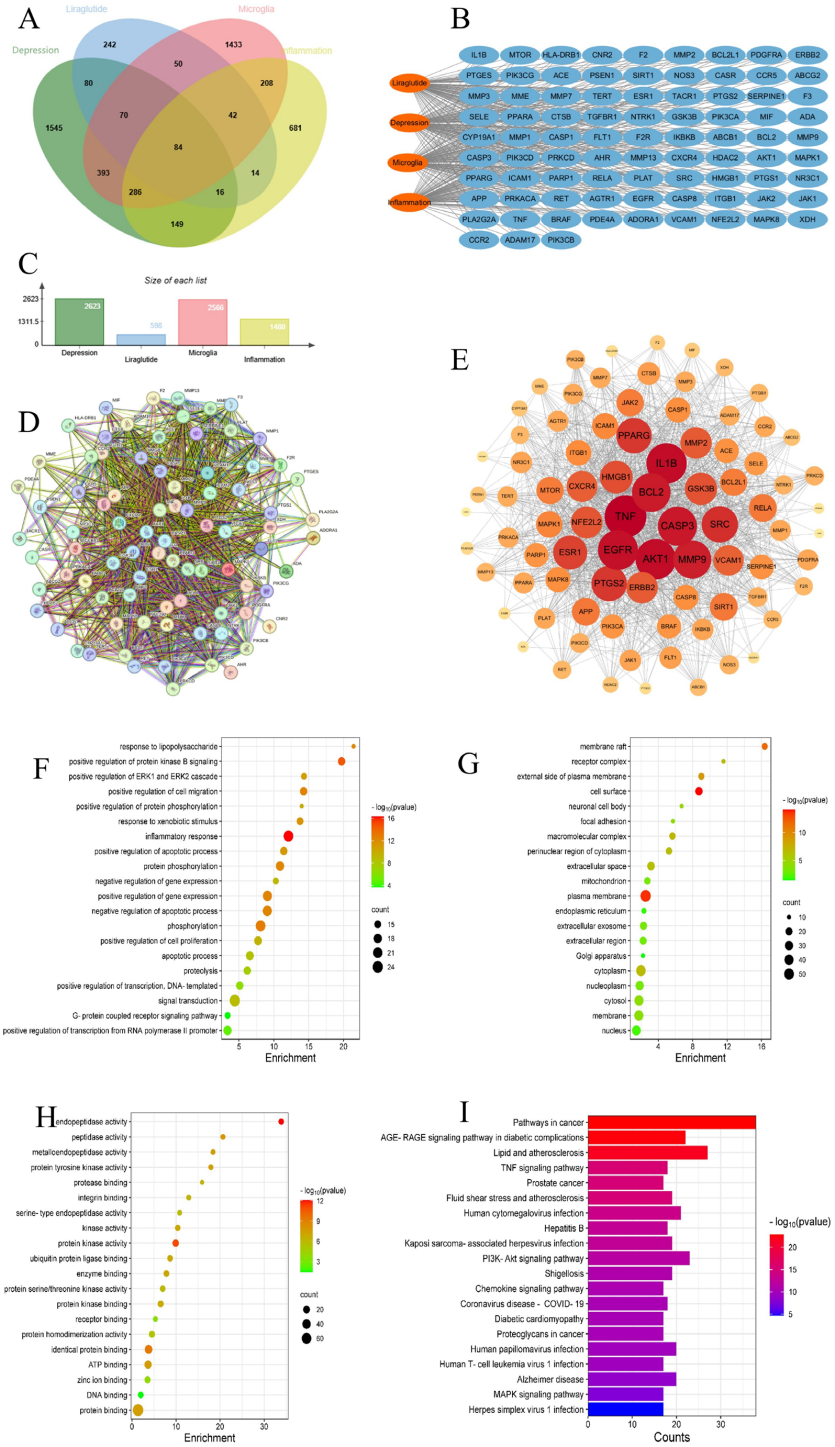
The network pharmacology analysis of Lir in the treatment of depression. (A) Venn diagram showing the overlapping targets between Lir‐related targets and depression‐related targets. (B) Drug–target–disease interaction network constructed using Cytoscape, illustrating the potential targets through which Lir may exert antidepressant effects. (C) Number of targets. (D) The PPI network of genes for Lir treatment of depression by the STRING database. (E) The core targets in the PPI network obtained using the CytoHubba plugin. (F) The top 20 biological processes (BPs). (G) The top 20 cellular components (CCs) (H) The top 20 molecular functions (MFs). (I) The top 20 KEGG signalling pathways.

We obtained 598 unique targets for Lir from databases, such as SEA, The Swiss Target Prediction and TargetNet. Furthermore, from databases including GeneCards, OMIM and CTD, a compilation of 2623 depression‐related targets, 2566 microglial cell targets and 1480 inflammation‐related targets was obtained, post‐deduplication. Importing these datasets into the Venn online tool identified 84 common targets, as depicted in Figure 1A, suggesting their potential relevance in Lir's therapeutic approach to depression. Figure 1C illustrates the distribution of depression, liraglutide, microglial cell and inflammation targets across different databases. Subsequently, these 84 common targets were inputted into the STRING database, generating a PPI network graph with 84 nodes and 1337 edges (Figure 1D). We then imported the data into Cytoscape software for visualisation analysis, as shown in Figure 1B, to generate the drug‐target‐disease network. The nodes were sorted based on their degree values, and the top 20 core targets selected from Figure 1E, depicted in Table S2, highlight inflammation factors, apoptosis‐related proteins, NFE2L2 and HMGB1, among others. Combined with previous relevant studies suggesting that these core proteins may play a crucial role in GLP‐1RA's treatment of depression linked to neuroinflammation involving microglial cells.

#### 3.1.2 GO Biological Function and KEGG Pathway Enrichment Analysis

Potential targets for Lir in the treatment of depression were analysed for GO biological function enrichment and KEGG pathway enrichment using the DAVID database. The GO biological function enrichment analysis identified 550 BPs, 74 CCs and 87 MFs involving the 84 common targets.

As shown in the bubble charts of Figure 1F–H, the top 20 genes in each of the three categories were selected. The targets are involved in biological processes, such as response to LPS, inflammatory response, positive regulation of the apoptotic process and protein phosphorylation, involving cellular components, such as the cell surface and perinuclear region of the cytoplasm, and molecular functions including endopeptidase activity, peptidase activity and metalloendopeptidase activity. The KEGG pathway enrichment analysis identified 164 signalling pathways, with the top 20 significant pathways shown in Figure 1I. Notable pathways include the pathways in cancer, TNF signalling pathway and PI3K‐Akt signalling pathway. In summary, Lir may regulate depression related to neuroinflammation involving microglial cells through these pathways.

### 3.2 Antidepressant Effects of Lir

#### 3.2.1 Effect of Lir on Depression‐Like Behaviours and Serum Cortisol (CORT) Levels in Mice

**FIGURE 2:**
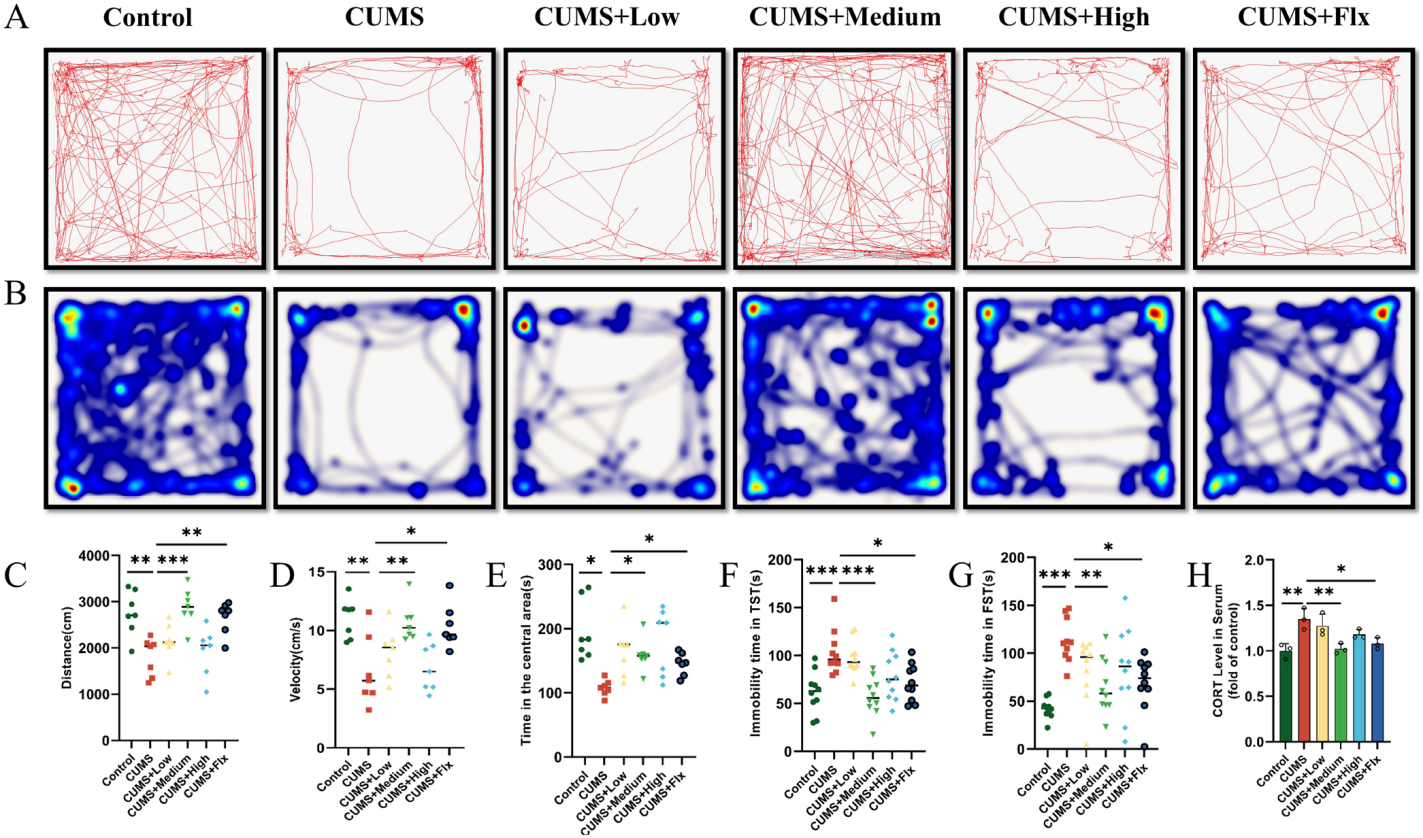
Effect of Lir on depression‐like behaviours and serum CORT levels in mice. (A) Representative OFT movement trajectory maps showing the exploratory behaviour and locomotor activity of mice (*n* = 7). (B) Heat map of movements in the OFT (*n* = 7). (C–E) The total distance travelled, movement speed and time spent in the centre area within 5 min for each group of mice (*n* = 7). (F) Immobility time in the TST, indicative of behavioural despair (*n* = 10). (G) Immobility time in the FST, another classical index of depression‐like behaviour (*n* = 10). (H) Serum CORT levels as a physiological marker of stress response (*n* = 3). Normally distributed data were analysed by one‐way ANOVA with Bonferroni or Tamhane's T2 post hoc tests, whereas non‐normally distributed data were assessed using the Kruskal‐Wallis test. Data are presented as mean ± SEM. *p < 0.05, **p < 0.01, ***p < 0.001.

We established the CUMS depression model in mice as previously described, and assessed spontaneous activity using the OFT. The results show that CUMS effectively reduced spontaneous activity in mice compared to the Control group (Figure 2A–E). To determine the effect of different doses of Lir on depressive‐like behaviour in CUMS‐exposed mice, we studied the therapeutic effects of 50, 150 and 300 μg/kg/day Lir using the TST and FST. The results revealed that (Figure 2F,G), compared to the CUMS group, the CUMS + medium‐dose Lir group showed significantly reduced immobility time in both TST (*p* < 0.001) and FST (*p =* 0.01), with antidepressant effects similar to Flx (TST: *p* = 0.023; FST: *p* = 0.022).

Depression patients often exhibit HPA axis hyperactivity, leading to increased CORT levels, which is mirrored by increased serum CORT in animal models. Serum CORT levels in mice were measured using ELISA. As shown in Figure 2H, CUMS significantly increased serum CORT levels, which decreased following intraperitoneal injection of medium‐dose Lir (*p =* 0.01) and Flx (*p =* 0.04).

The results indicate that Lir effectively alleviates anxiety‐ and depression‐like behaviours and reduces serum CORT levels in CUMS‐exposed mice. The medium dose of Lir demonstrated the most effective results; therefore, we will use this dose for subsequent mechanistic studies.

#### 3.2.2 Lir Can Reduce Neuronal Damage in the Brains of Mice

**FIGURE 3:**
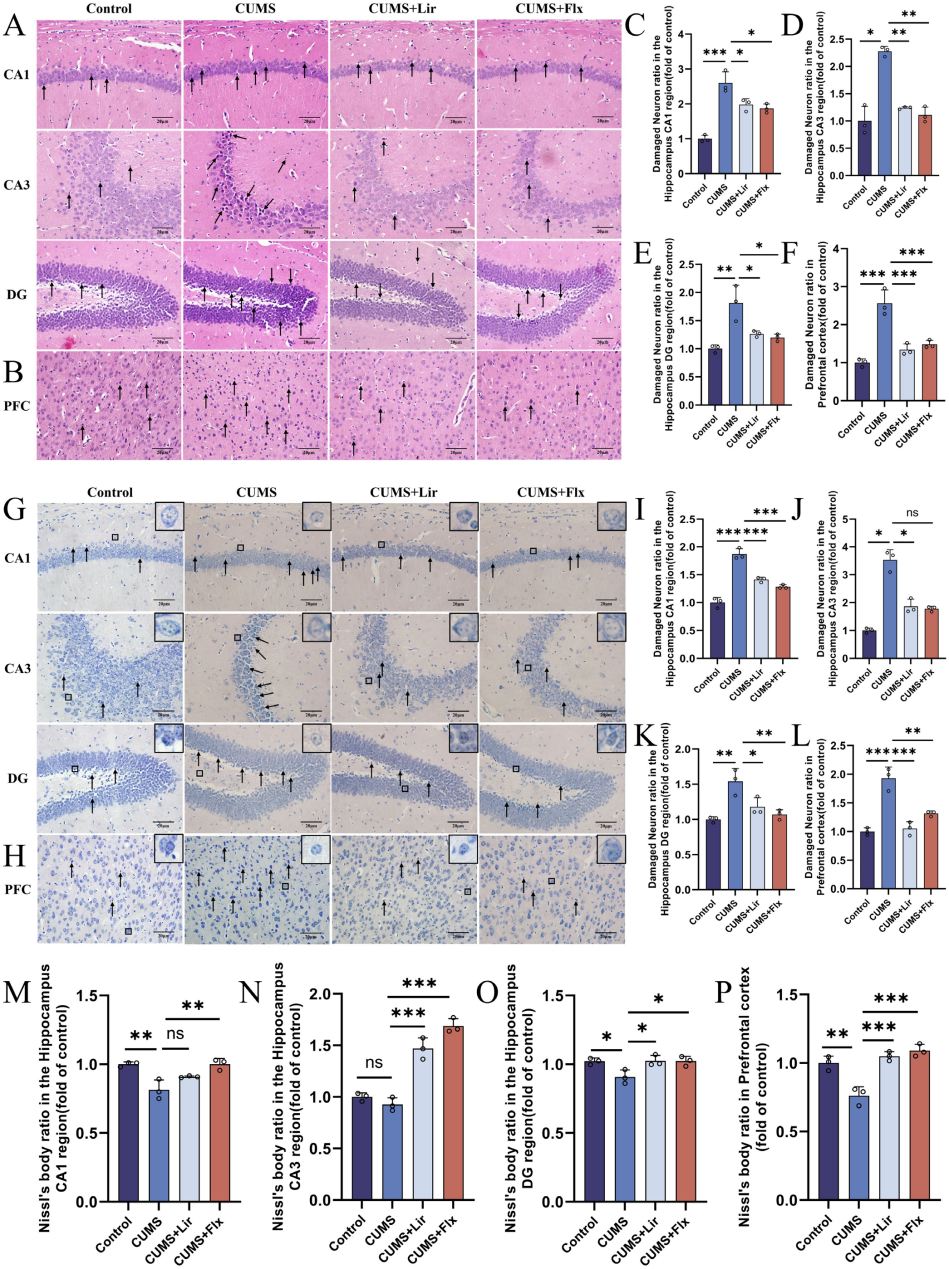
The neuroprotective effects of Lir on brain neurons in mice. Representative histopathological images of the HIP (A) and PFC (B) were obtained using H&E staining. Neuronal damage was assessed in specific hippocampal regions: CA1 (C), CA3 (D) and DG (E), as well as in the PFC (F), across different experimental groups. Nissl staining of HIP (G) and PFC (H) tissues further corroborated these findings. Quantitative analysis revealed neuronal integrity in HIP CA1 (I), CA3 (J), DG (K) regions and in PFC (L) across experimental groups. Nissl body counts in HIP CA1 (M), CA3 (N), DG (O) and PFC (P) regions were also quantified (*n* = 3). Scale bar = 20 μm. The arrows indicate damaged neurons, and the zoomed views highlight the cells containing Nissl bodies. Normally distributed data were analysed by one‐way ANOVA with Bonferroni or Tamhane's T2 post hoc tests, whereas non‐normally distributed data were assessed using the Kruskal‐Wallis test. Data are presented as mean ± SEM. *p < 0.05, **p < 0.01, ***p < 0.001.

Following behavioural experiments, we used H&E and Nissl staining to stain tissue from the HIP and PFC to observe changes in neuronal morphology and structure. In H&E staining (Figure 3A,B), CUMS stimulation resulted in neuronal damage characterised by sparse cell arrangement, nuclear condensation and disappearance of nucleoli. Compared to the CUMS group (Figure 3C–F), treatment with Lir showed a significant reduction in the number of damaged neurons in HIP and PFC regions.

In Nissl staining, Nissl bodies appeared purple‐blue, cell nuclei were light blue, and the background was colourless. Results (Figure 3G,H) revealed that compared to the CUMS group (Figure 3I–P), the Lir treatment group showed rounder nuclei, clearer nucleoli and fewer damaged neurons in HIP and PFC regions. In the CUMS group, Nissl bodies were concentrated at the edges and reduced in content within the cytoplasm. Conversely, the Lir treatment group showed increased Nissl bodies within the cell bodies in HIP and PFC regions.

These findings demonstrate that Lir exhibits a protective effect against neuronal damage in the HIP and PFC regions of depressive mice.

#### 3.2.3 Lir Inhibits the Activation of Microglia in the Brains of Mice

**FIGURE 4:**
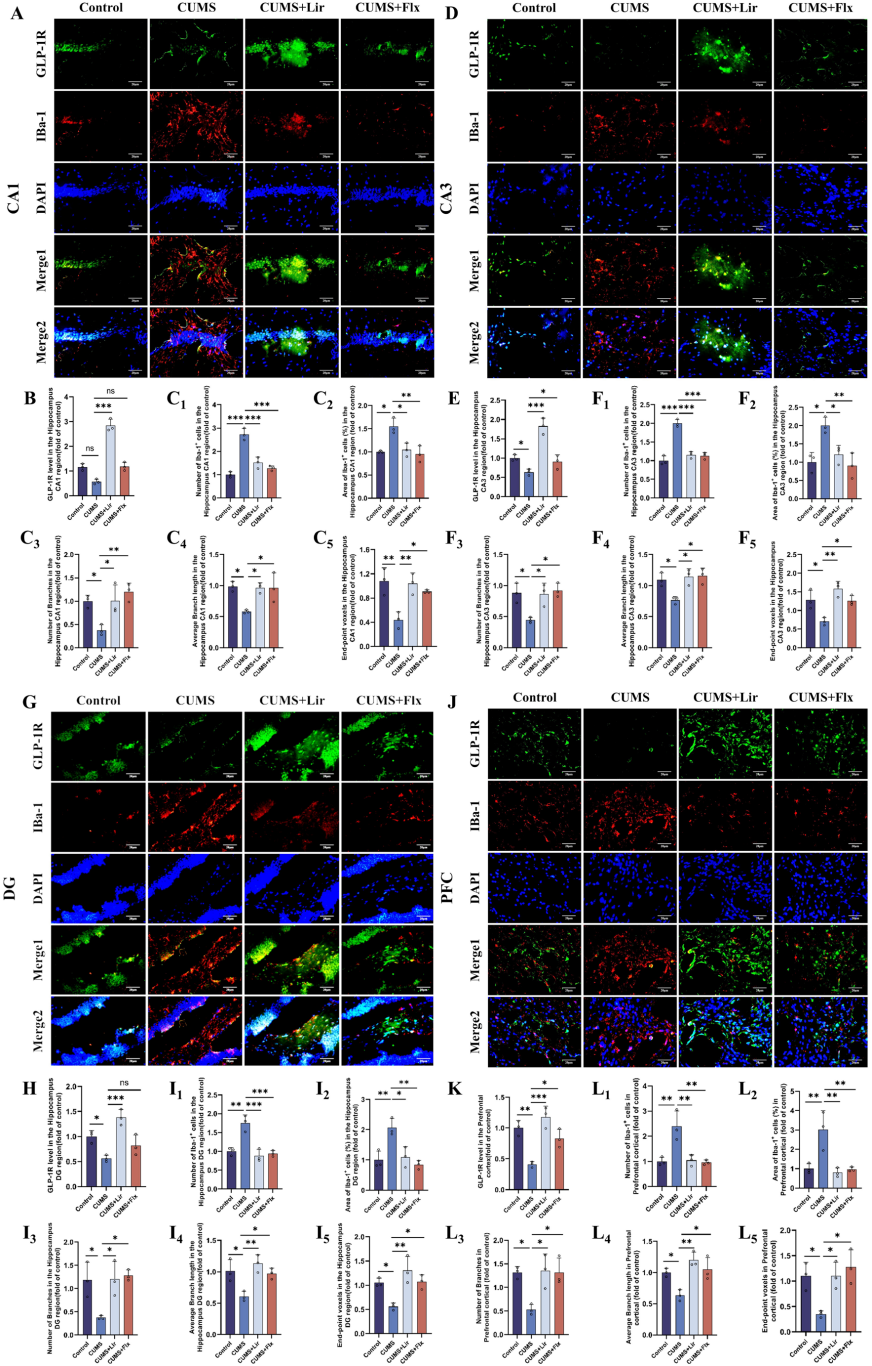
Lir reduced microglial activation in the HIP and PFC regions of CUMS‐induced depressed mice. Representative immunofluorescence images showing GLP‐1R (red) and microglial (IBa‐1, green) co‐staining in the HIP (A, D, G) and PFC (J) regions. Quantitative analysis of GLP‐1R expression levels in the HIP (B, E, H) and PFC (K) was performed for each experimental group. The number and total area of microglia were assessed in the HIP (C1‐C2, F1‐F2, I1‐I2) and PFC (L1‐L2) regions. Microglial morphological complexity was evaluated by analysing the branches in the HIP (C3‐C4, F3‐F4, I3‐I4) and PFC (L3‐L4), and the end‐point voxels was quantified in the HIP (C5, F5, I5) and PFC (L5). (*n* = 3). Scale bar = 20 μm. Normally distributed data were analysed by one‐way ANOVA with Bonferroni or Tamhane's T2 post hoc tests, whereas non‐normally distributed data were assessed using the Kruskal‐Wallis test. Data are presented as mean ± SEM. *p < 0.05, **p < 0.01, ***p < 0.001.

To explore the effects of Lir on microglia, we used immunofluorescence staining to detect the expression of GLP‐1R and the microglial marker IBa‐1 in the HIP (Figure 4A–I) and PFC (Figure 4J–L) regions of the mice. It can be seen that compared to the CUMS group, treatment with Lir increased the expression of GLP‐1R in the HIP and PFC regions. The administration of Flx shows the same therapeutic effects as Lir.

Previous studies [20] have shown that the morphology of microglia typically changes according to their functional state. In the resting state, microglia have long, thin branches used to monitor the microenvironment. During depression, microglia enlarge, and their branches become shorter and thicker, sometimes transforming into an amoeboid shape with phagocytic functions. As shown in Figure 4, CUMS increased the number and area of microglia in brain regions of mice, reduced the number of end‐point voxels, and decreased the number of branches and the average length of branches. However, with the increased expression of GLP‐1R, some of these CUMS‐induced changes were partially reversed.

Previous studies have demonstrated that CD68 is an important marker of activated microglia [30]. Therefore, we assessed microglial activation by co‐localising IBa‐1 and CD68. As shown in Figure S3, microglia in the HIP and PFC of CUMS‐induced depressed mice exhibited enlarged and thickened cell bodies, along with increased area ratios of IBa‐1 and CD68. These alterations were partially reversed following Lir treatment.

These results indicate that Lir may alleviate CUMS‐induced neuroinflammation by inhibiting the activation of microglia in the HIP and PFC regions of mice.

#### 3.2.4 Effects of Lir on Nrf2 and HMGB1 Protein Levels, Oxidative Stress Factors and Inflammatory Factors in CUMS‐Exposed Mice

We examined the expression levels of Nrf2 and HMGB1 proteins in the HIP and PFC tissues of each experimental group, along with oxidative stress and inflammation‐related factors.

**FIGURE 5:**
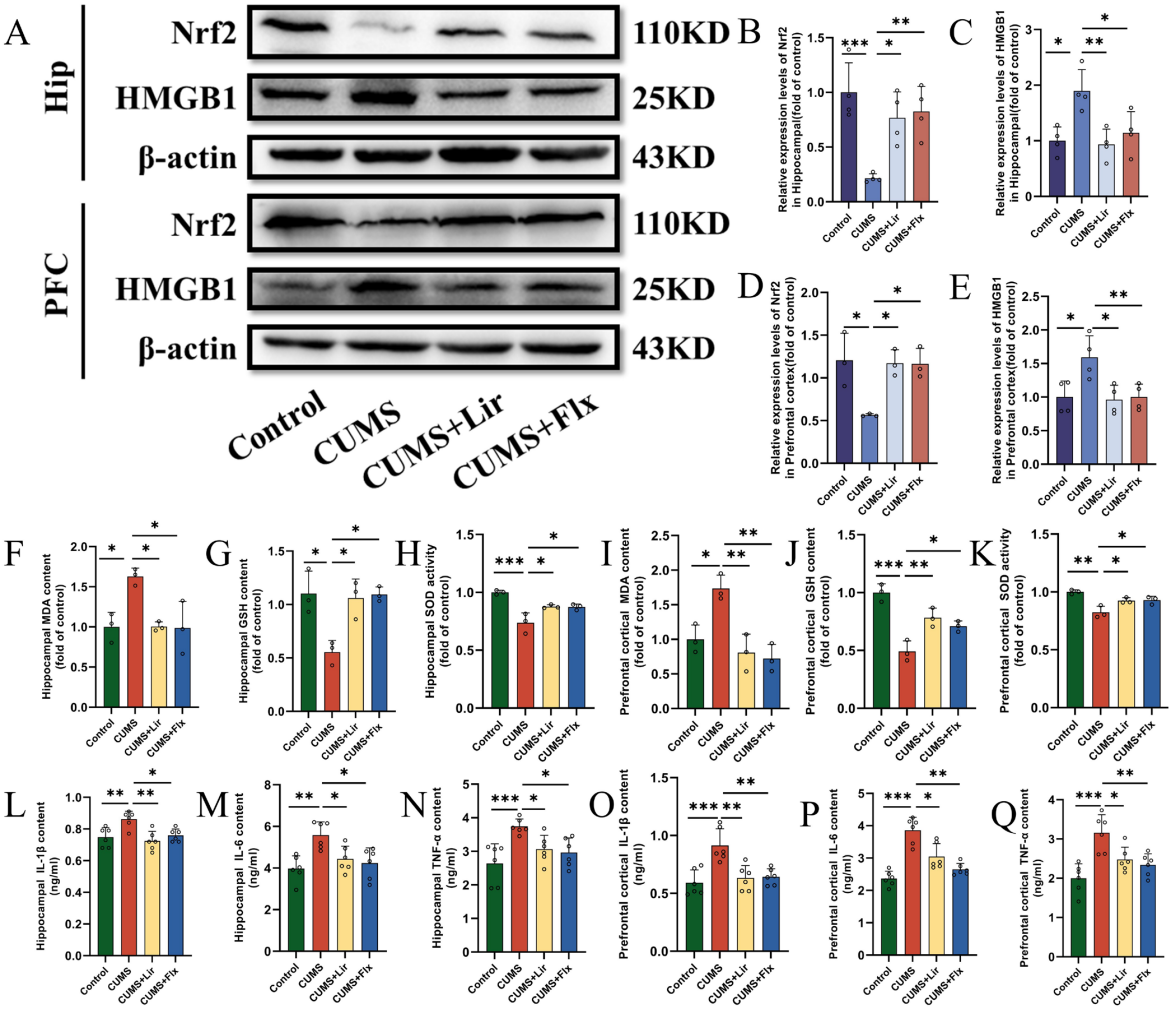
Effects of Lir on Nrf2 and HMGB1 protein expression levels, oxidative stress and inflammatory factors in the HIP and PFC tissues. (A) Representative WB images showing the expression levels of Nrf2 and HMGB1 proteins. Quantitative analysis of Nrf2 (B) and HMGB1 (C) protein levels in HIP tissues across all experimental groups. Quantitative analysis of Nrf2 (D) and HMGB1 (E) protein levels in PFC tissues. Oxidative stress factors in HIP tissues, including MDA (F), GSH (G) and SOD (H) levels. Oxidative stress factors in PFC tissues, including MDA (I), GSH (J) and SOD (K) levels. Levels of inflammatory factors including IL‐1β (L), IL‐6 (M) and TNF‐α (N) in HIP tissues, and IL‐1β (O), IL‐6 (P) and TNF‐α (Q) in PFC tissues. (*n* = 3–6). Normally distributed data were analysed by one‐way ANOVA with Bonferroni or Tamhane's T2 post hoc tests, whereas non‐normally distributed data were assessed using the Kruskal–Wallis test. Data are presented as mean ± SEM. *p < 0.05, **p < 0.01, ***p < 0.001.

WB analysis (Figure 5A) showed that CUMS stimulation reduced Nrf2 protein level in HIP (Figure 5B) and PFC (Figure 5D) tissues while increasing HMGB1 protein level in HIP (Figure 5C) and PFC (Figure 5E) tissues. Lir treatment increased Nrf2 protein level in HIP and PFC tissues (HIP [*p =* 0.021], PFC [*p =* 0.045]) and decreased HMGB1 protein level (HIP [*p =* 0.008], PFC [*p =* 0.031]), showing effects similar to the antidepressant Flx.

Oxidative stress damage and neuroinflammation are critical pathogenic factors in depression. We measured the levels of oxidative stress factors MDA (Figure 5F,I), GSH (Figure 5G,J), SOD (Figure 5H,K) and inflammatory factors IL‐1β (Figure 5L,O), IL‐6 (Figure 5M,P) and TNF‐α (Figure 5N,Q) in the HIP and PFC tissues of each group. MDA levels increased under CUMS stimulation in both brain regions, but this change was reversed by Lir treatment. In contrast, GSH levels and SOD activity were higher in both brain regions under Lir and Flx treatments compared to the CUMS group. CUMS stimulation increased levels of inflammatory factors IL‐1β, IL‐6 and TNF‐α in both brain regions, whereas Lir and Flx treatments reduced these levels.

These results indicate that Lir can increase Nrf2 and decrease HMGB1 protein levels, demonstrating efficacy in antioxidative stress and anti‐inflammatory activities.

### 3.3 Lir Increases Nrf2 Protein Levels and Promotes Its Nuclear Translocation Following CUMS Exposure

**FIGURE 6:**
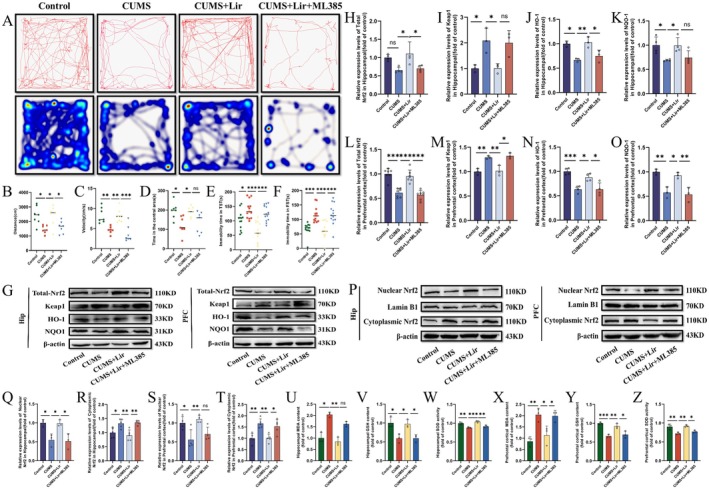
Effects of Lir on Nrf2 expression and oxidative stress following CUMS exposure. (A) Representative movement trajectory and activity heat maps of mice in the OFT. (B) Distance travelled (*n* = 7). (C) Movement speed (*n* = 7). (D) Time spent in the central area (*n* = 7). (E) Immobility time in TST (*n* = 13). (F) Immobility time in FST (*n* = 13). (G) Representative WB images of total protein expression in HIP and PFC tissues, showing (H) Nrf2, (I) Keap1, (J) HO‐1 and (K) NQO1 expression in HIP and (L) Nrf2, (M) Keap1, (N) HO‐1 and (O) NQO1 expression in PFC. (P) Representative WB images for nuclear and cytoplasmic fractions of Nrf2 in HIP and PFC tissues. Quantification of Nrf2 levels in the nuclear (Q, S) and cytoplasmic (R, T) compartments of HIP and PFC tissues. Levels of oxidative stress factors including MDA (U), GSH (V), SOD (W) in HIP tissue and MDA (X), GSH (Y) and SOD (Z) in PFC tissue (*n* = 3–5). Normally distributed data were analysed by one‐way ANOVA with Bonferroni or Tamhane's T2 post hoc tests, whereas non‐normally distributed data were assessed using the Kruskal–Wallis test. Data are presented as mean ± SEM. *p < 0.05, **p < 0.01, ***p < 0.001.

Given the importance of Nrf2 in Lir treatment for depression, we used the Nrf2 inhibitor ML385 to suppress Nrf2 expression in mice. Compared to the CUMS group, Lir treatment increased spontaneous activity in the OFT (Figure 6A–D) and reduced immobility time in both the TST (Figure 6E) and FST (Figure 6F), while ML385 reverses the therapeutic effect of Lir.

Subsequently, we explored the protein levels of Nrf2 and its downstream proteins Keap1, HO‐1 and NQO1 in the HIP and PFC tissues (Figure 6G). The results (Figure 6H–O) indicate that, compared to the CUMS group, Lir treatment significantly increased the protein levels of Nrf2 (HIP [*p =* 0.022], PFC [*p* < 0.001]), HO‐1 (HIP [*p =* 0.006], PFC [*p =* 0.014]) and NQO1 (HIP [*p =* 0.05], PFC [*p =* 0.017]), whereas reducing the Keap1 protein level (HIP [*p =* 0.038], PFC [*p =* 0.01]). ML385 eliminated Lir's effects on these proteins.

The main mechanism of Nrf2 is the rapid release from Keap1 into the nucleus, resulting in increased levels of downstream proteins to regulate the expression of MDA, GSH and SOD during oxidative stress. Therefore, we measured Nrf2 levels in the nucleus and cytoplasm of both HIP and PFC tissues (Figure 6P). Compared to the CUMS group (Figure 6Q–T), Lir can promote the transfer of Nrf2 to nuclear in both brain regions and reduce its cytoplasmic expression, whereas ML385 reverses this process.

Next, we measured oxidative stress factors in HIP and PFC tissues. The results (Figure 6U–Z) show that CUMS stimulation increased MDA levels and decreased GSH levels and SOD activity, whereas Lir treatment reversed these changes, and ML385 inhibited Nrf2's antioxidative effects.

In conclusion, the antioxidative stress effects of Lir are likely related to the activation of the Nrf2 protein.

### 3.4 Lir Can Attenuate the Pro‐Inflammatory Effects of HMGB1 in Depression

**FIGURE 7:**
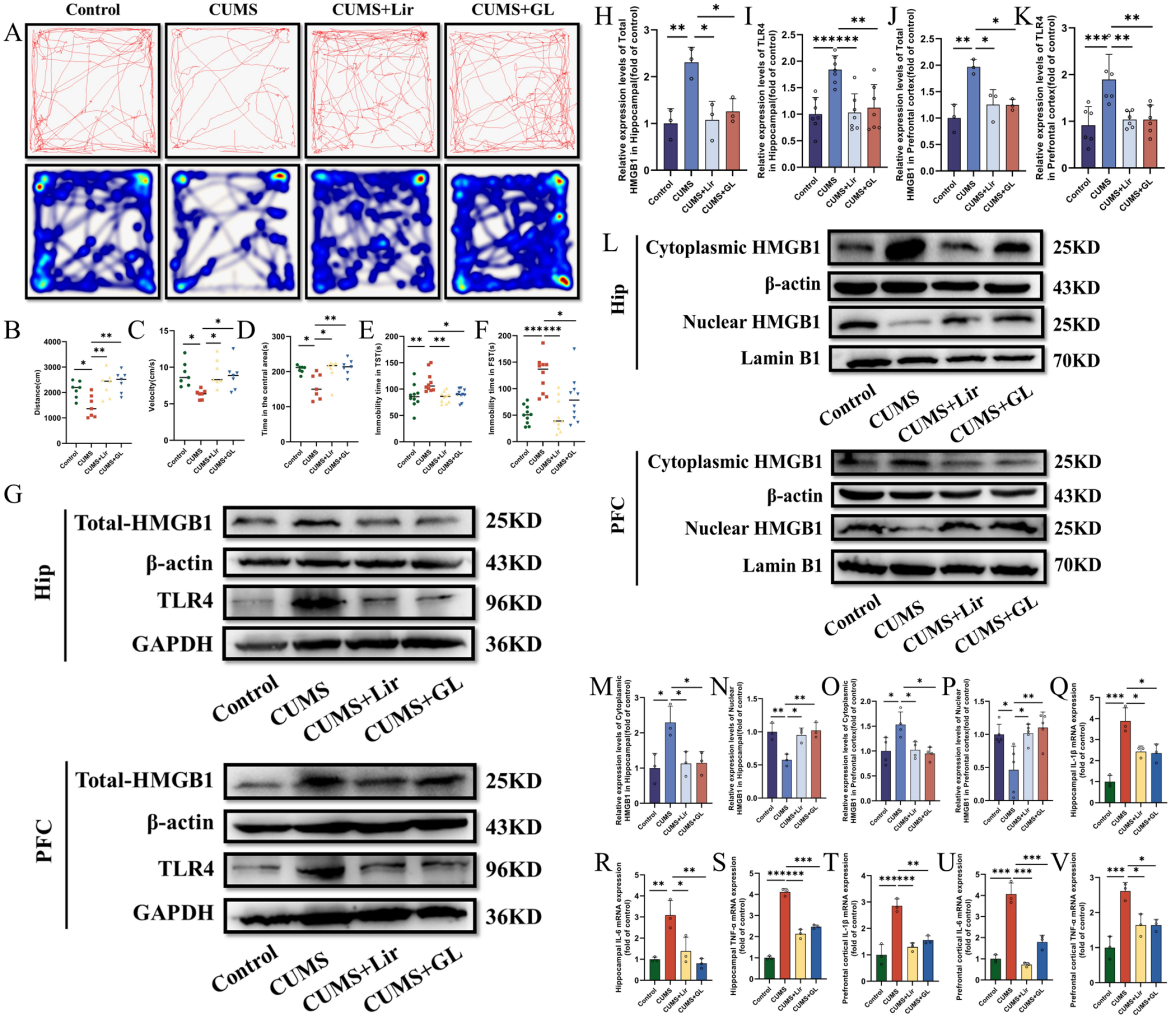
The inhibitory effect of Lir on HMGB1 expression and related behavioural improvements in CUMS‐exposed mice. (A) Representative movement trajectory and activity heat maps of mice in the OFT. (B) Movement distance (*n* = 7). (C) Movement speed (*n* = 7). (D) Time spent in the central area (*n* = 7). (E) Immobility time in the TST (*n* = 10). (F) Immobility time in the FST (*n* = 10). (G) Representative WB images showing protein expression. Quantification of total HMGB1 (H) and TLR4 (I) protein levels in HIP tissue and total HMGB1 (J) and TLR4 (K) levels in PFC tissue. (L) Representative WB images. Quantification of HMGB1 protein levels in the cytoplasmic and nuclear fractions of the HIP (M, N) and PFC (O, P) tissue (*n* = 3–5). Levels of inflammatory cytokines IL‐1β (Q), IL‐6(R), TNF‐α (S) in HIP tissue and IL‐1β (T), IL‐6(U), TNF‐α (V) in PFC tissue (*n* = 3). Normally distributed data were analysed by one‐way ANOVA with Bonferroni or Tamhane's T2 post hoc tests, while non‐normally distributed data were assessed using the Kruskal–Wallis test. Data are presented as mean ± SEM. *p < 0.05, **p < 0.01, ***p < 0.001.

In this study, we verified the pro‐inflammatory effects of HMGB1 using the HMGB1 inhibitor Glycyrrhizin (GL). Compared to the CUMS group, treatment with Lir and GL increased spontaneous activity in the OFT (Figure 7A–D) and reduced immobility time in the TST (Figure 7E) and FST (Figure 7F).

Next, we investigated the protein levels of HMGB1 and its downstream protein TLR4 in the HIP and PFC of various groups of mice (Figure 7G). The results (Figure 7H–K) showed that, compared to the CUMS group, treatment with Lir significantly reduced the protein levels of HMGB1 (HIP [*p =* 0.011], PFC [*p =* 0.02]) and TLR4 (HIP [*p =* 0.001], PFC [*p =* 0.005]) and GL treatment exhibited similar effects. Additionally, we examined the protein level of HMGB1 in the nucleus and cytoplasm (Figure 7L). It was observed that CUMS increased the cytoplasmic protein level of HMGB1 in HIP and PFC tissues (Figure 7M,O) and reduced its nuclear expression (Figure 7N,P). Compared to the CUMS group, Lir treatment decreased the cytoplasmic protein level of HMGB1 (HIP [*p =* 0.039], PFC [*p =* 0.032]) and increased the nuclear protein level (HIP [*p =* 0.021], PFC [*p =* 0.019]) and GL showed similar therapeutic effects. Furthermore, we assessed the mRNA expression levels of inflammatory cytokines (Figure 7Q–V). It was evident that treatment with Lir and GL reduced the expression of inflammatory factors.

The above results indicate that Lir can decrease the expression of HMGB1 in the HIP and PFC regions of CUMS‐exposed mice, thereby alleviating the occurrence of neuroinflammation.

### 3.5 Effect of Different Concentrations of LPS and Lir on BV2 Cell Viability

**FIGURE 8:**
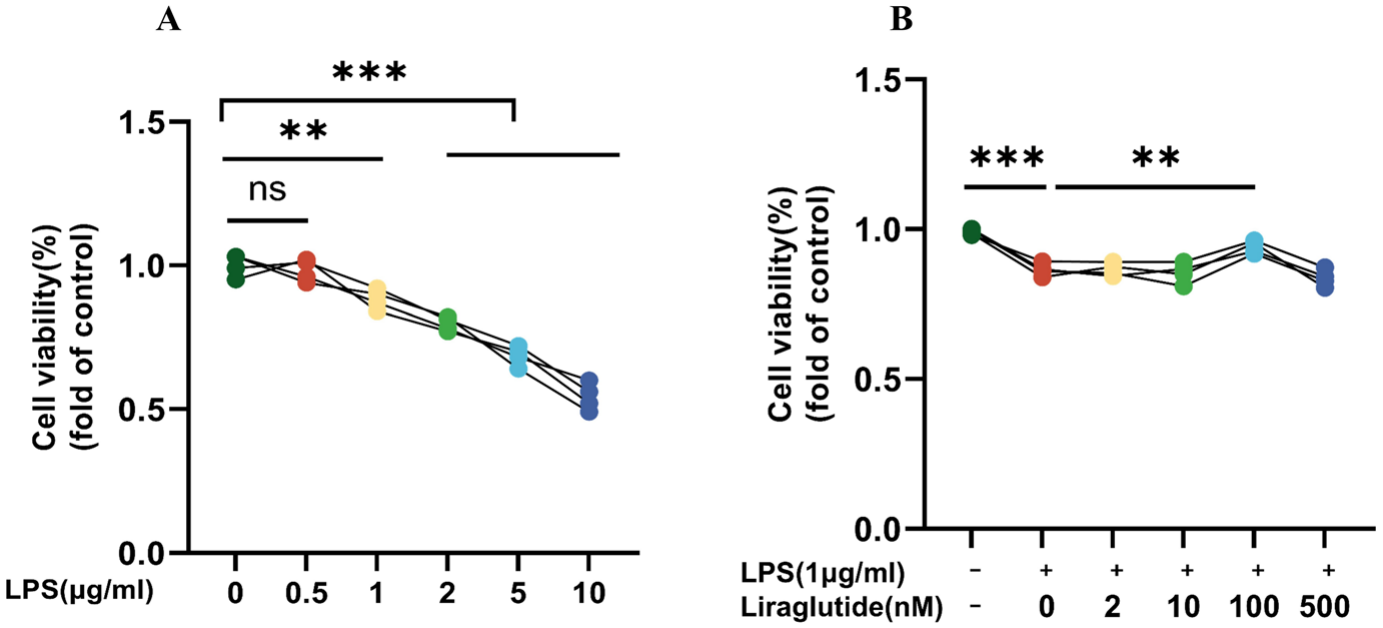
Statistical analysis of BV2 cell viability assessed by CCK‐8 assay. (A) Effect of different concentrations of LPS on the viability of BV2 microglial cells. (B) Effect of varying concentrations of Lir on LPS‐stimulated BV2 cells, showing the potential protective effect of Lir against LPS‐induced reduction in cell viability (*n* = 4). Normally distributed data were analysed by one‐way ANOVA with Bonferroni or Tamhane's T2 post hoc tests, whereas non‐normally distributed data were assessed using the Kruskal–Wallis test. Data are presented as mean ± SEM. *p < 0.05, **p < 0.01, ***p < 0.001.

First, we stimulated BV2 cells with different concentrations of LPS (0, 0.5, 1, 2, 5 and 10 μg/mL) to establish a depression model. The results (Figure 8A) showed that higher concentrations of LPS (2, 5 and 10 μg/mL) significantly reduced cell viability compared to 1 μg/mL. Therefore, we selected 1 μg/mL LPS for stimulating BV2 cells. To determine the appropriate concentration of Lir for treating the LPS‐induced depression model, various concentrations of Lir (0, 2, 10, 100, 500 nM) were added to LPS‐stimulated BV2 cells. The results (Figure 8B) showed that, compared to the control group, LPS stimulation significantly reduced cell viability (*p* < 0.001). There was no significant difference in cell viability between the Lir‐treated groups (2, 10, 500 nM) and the LPS group. In contrast, 100 nM Lir (*p =* 0.004) increased cell viability in the LPS group. Therefore, 100 nM Lir was selected for use in subsequent experiments.

### 3.6 The Regulatory Role of Nrf2 on HMGB1 During the Antidepressant Effect of Lir

**FIGURE 9:**
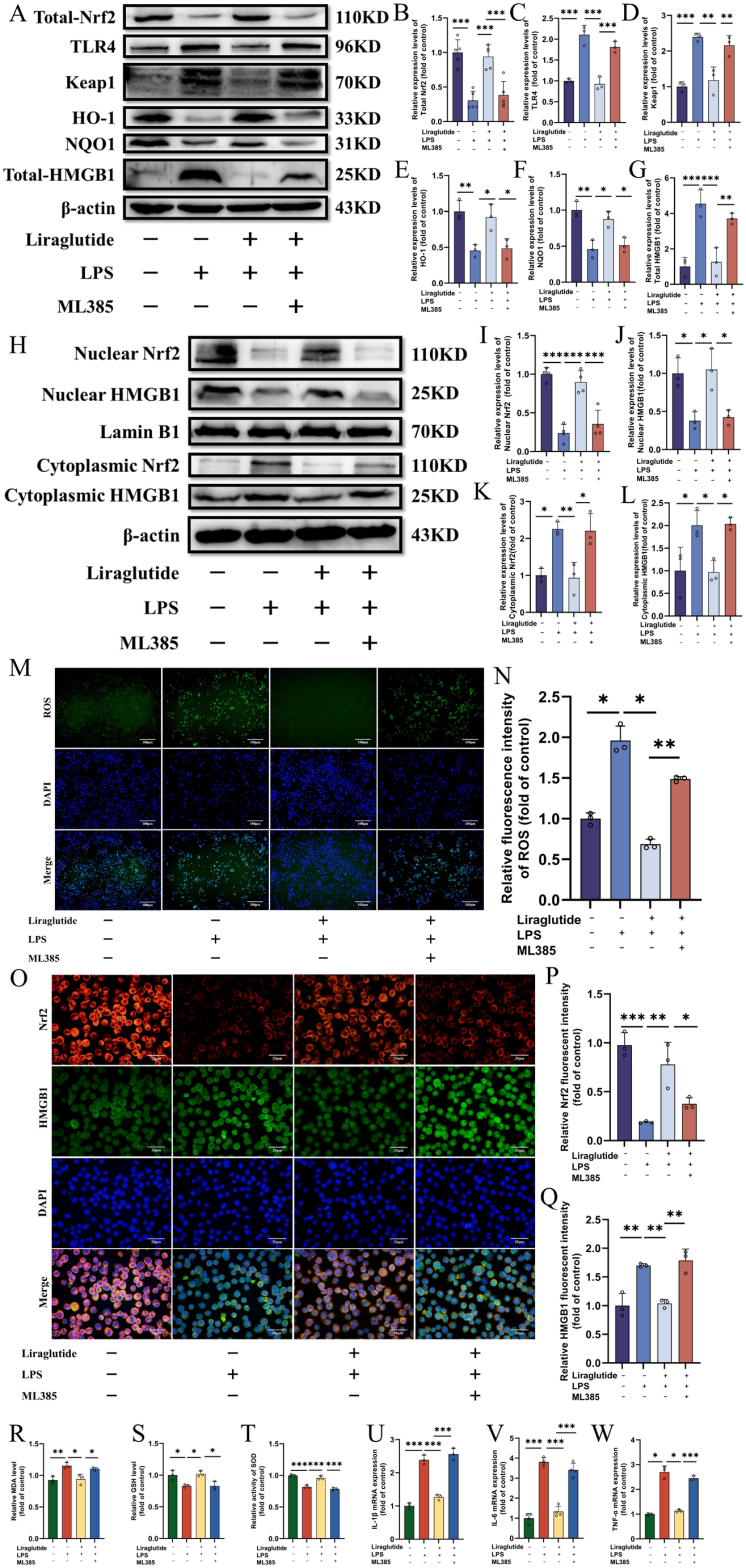
Representative WB images showing the expression of proteins involved in the Nrf2 and HMGB1 signalling pathways. (A) Representative WB images showing the expression of related proteins of Nrf2 and HMGB1 signalling pathways. Quantitative analysis of protein expression levels for Nrf2 (B), keap1 (D), HO‐1 (E), NQO1 (F), HMGB1 (G) and TLR4 (C). (H) Representative WB images. Expression of Nuclear Nrf2 (I), Nuclear HMGB1 (J), Cytoplasmic Nrf2 (K) and Cytoplasmic HMGB1 (L). (M) Detection of ROS by immunofluorescence. (N) Statistical analysis of ROS fluorescence intensity, scale bar = 100 μm. (O) Representative immunofluorescence images showing colocalisation of Nrf2 and HMGB1 in BV2 cells, scale bar = 20 μm. Quantification of fluorescence intensity for Nrf2 (P) and HMGB1 (Q). Levels of oxidative stress markers MDA (R), GSH (S) and SOD (T), as well as inflammatory cytokines IL‐1β (U), IL‐6 (V) and TNF‐α (W) (*n* = 3–5). Normally distributed data were analysed by one‐way ANOVA with Bonferroni or Tamhane's T2 post hoc tests, while non‐normally distributed data were assessed using the Kruskal‐Wallis test. Data are presented as mean ± SEM. *p < 0.05, **p < 0.01, ***p < 0.001.

In this section, we explored the regulatory role of Nrf2 on HMGB1. Immunoblotting results showed that (Figure 9A), compared to the LPS group, Lir treatment promoted the expression of Nrf2 (Figure 9B, *p* < 0.001), HO‐1 (Figure 9E, *p =* 0.023) and NQO‐1 (Figure 9F, *p =* 0.012) at the protein level and inhibited the expression of keap1 (Figure 9D, *p =* 0.002). However, ML385 inhibited the effects of Lir. Subsequently, we examined the protein levels of HMGB1 and TLR4 proteins. The results indicated that Lir treatment reduced the high protein levels of HMGB1 (Figure 9G, *p =* 0.001) and TLR4 (Figure 9C, *p* < 0.001) induced by LPS. Compared to the LPS + Lir group, the Nrf2 inhibitor ML385 significantly increased the protein expression levels of HMGB1 and TLR4. Further evaluation of Nrf2 and HMGB1 protein levels in the nucleus and cytoplasm (Figure 9H–L) revealed that Lir promoted Nrf2 nuclear translocation (Figure 9I, *p* < 0.001) and inhibited cytoplasmic HMGB1 expression (Figure 9L, *p =* 0.035), whereas ML385 suppressed these effects.

Additionally, Lir reduced the mRNA expression of inflammatory cytokines IL‐1β (Figure 9U, *p* < 0.001), IL‐6 (Figure 9V, *p* < 0.001) and TNF‐α (Figure 9W, *p =* 0.031) in BV2 cells stimulated by LPS, whereas ML385 treatment diminished the reducing effect of Lir on these cytokines. Similarly, ML385 decreased the antioxidant stress capacity of Lir in LPS‐induced BV2 cells (Figure 9R–T).

Fluorescence detection of ROS (Figure 9M,N) showed that ROS fluorescence intensity was significantly higher in the LPS group compared to the control group (*p =* 0.031), and Lir treatment decreased ROS expression (*p =* 0.018). The addition of ML385 significantly increased ROS fluorescence intensity compared to the LPS + Lir group (*p =* 0.002).

In subsequent experiments, we used immunofluorescence to detect the expression of Nrf2 and HMGB1 proteins in BV2 cells. The results (Figure 9O) showed that, compared to the LPS group, Lir increased the Nrf2 protein level (Figure 9P, *p =* 0.004) while reducing HMGB1 expression (Figure 9Q, *p =* 0.004), and ML385 intervention reversed these effects. These results indicate that Lir exerts antioxidant and anti‐inflammatory effects by activating the Nrf2‐regulated HMGB1 pathway.

### 3.7 Exploring the Mechanistic Pathway of Lir in Activating Nrf2

**FIGURE 10:**
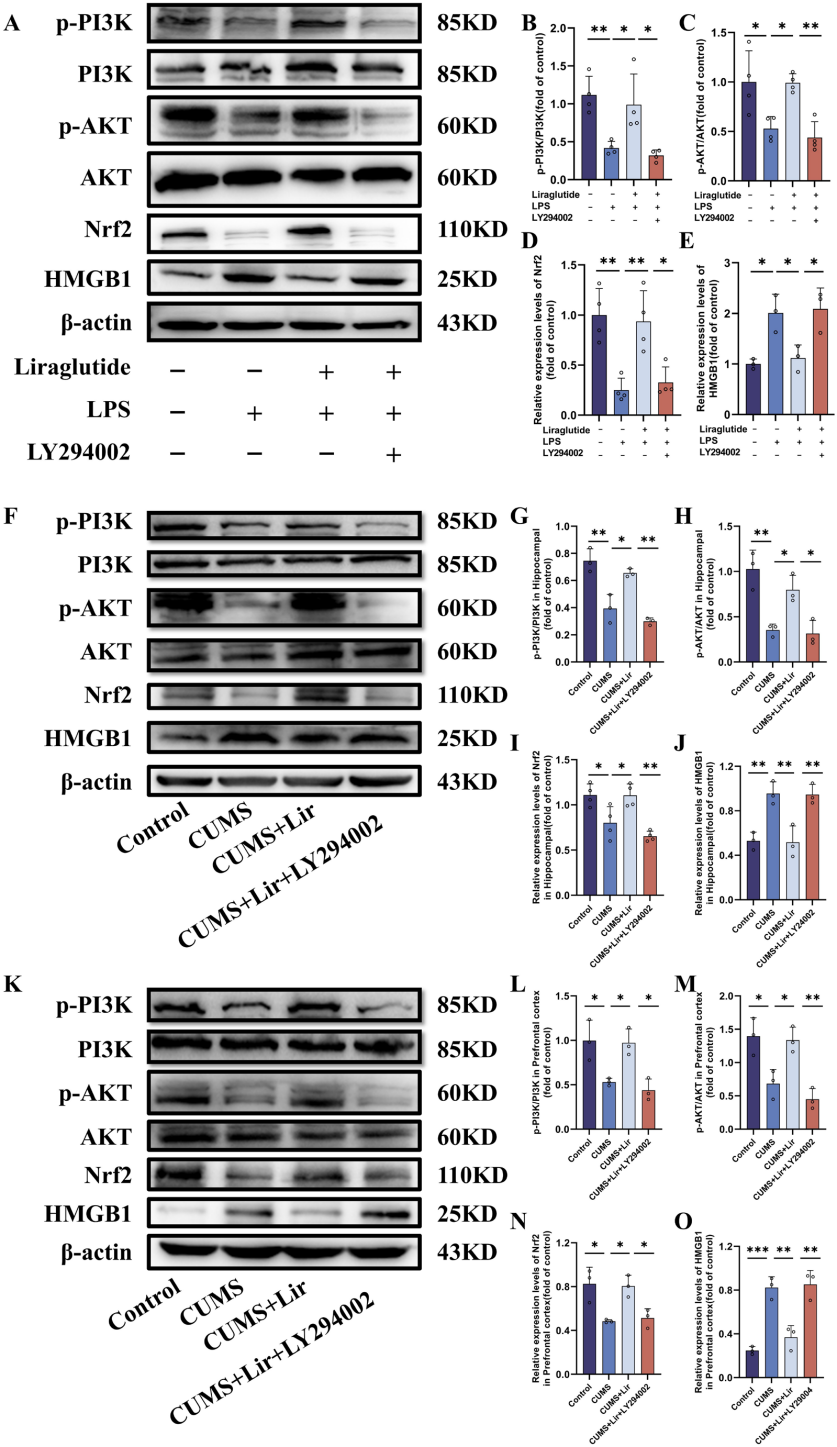
Investigation of the mechanistic pathway of Lir in the treatment of depression. (A) Representative WB images showing the expression of pathway‐related proteins in the in vitro experiment. Quantitative analysis of protein expression levels, including p‐PI3K/PI3K (B), p‐AKT/AKT (C), Nrf2 (D) and HMGB1 (E). (F, K) Representative WB images of pathway proteins in the HIP and PFC tissues of each group in the in vivo experiment. Quantitative analysis of protein expression levels in the HIP, including p‐PI3K/PI3K (G), p‐AKT/AKT (H), Nrf2 (I) and HMGB1 (J), and in the PFC, including p‐PI3K/PI3K (L), p‐AKT/AKT (M), Nrf2 (N) and HMGB1 (O) (*n* = 3–5). Normally distributed data were analysed by one‐way ANOVA with Bonferroni or Tamhane's T2 post hoc tests, whereas non‐normally distributed data were assessed using the Kruskal–Wallis test. Data are presented as mean ± SEM. *p < 0.05, **p < 0.01, ***p < 0.001.

To further investigate the potential mechanisms by which Lir activates Nrf2, we examined the activity of the PI3K/AKT pathway in BV2 cells. As shown in Figure 10A, this pathway enhances the stability and activity of Nrf2 through phosphorylation, thereby promoting the expression of downstream antioxidant genes. LPS inhibited the phosphorylation of PI3K (Figure 10B, *p* = 0.01) and AKT (Figure 10C, *p* = 0.028), and reduced the expression level of Nrf2 protein (Figure 10C, *p* = 0.003). However, Lir effectively reversed these changes. In addition, the PI3K pathway inhibitor LY294002 blocked the activation of the PI3K/AKT pathway by Lir, reduced Nrf2 protein expression, and subsequently increased the expression of HMGB1 protein.

Next, we validated this pathway in in vivo experiments (Figure 10F–O), and the results were consistent with those from the in vitro study. Exposure to CUMS inhibited the phosphorylation of PI3K and AKT proteins in the HIP (Figure 10F–J) and PFC (Figure 10K–O) regions of mice and reduced the expression level of Nrf2 protein. Lir largely reversed these changes. However, LY294002 blocked the activation effect of Lir on the PI3K/AKT pathway, leading to a decrease in Nrf2 expression and an increase in HMGB1 protein levels.

These findings further confirm that Lir can activate Nrf2 through the PI3K/AKT pathway, thereby suppressing HMGB1 expression and the inflammatory response induced by the depression model.

### 3.8 Neuroprotective Effects of Lir

**FIGURE 11:**
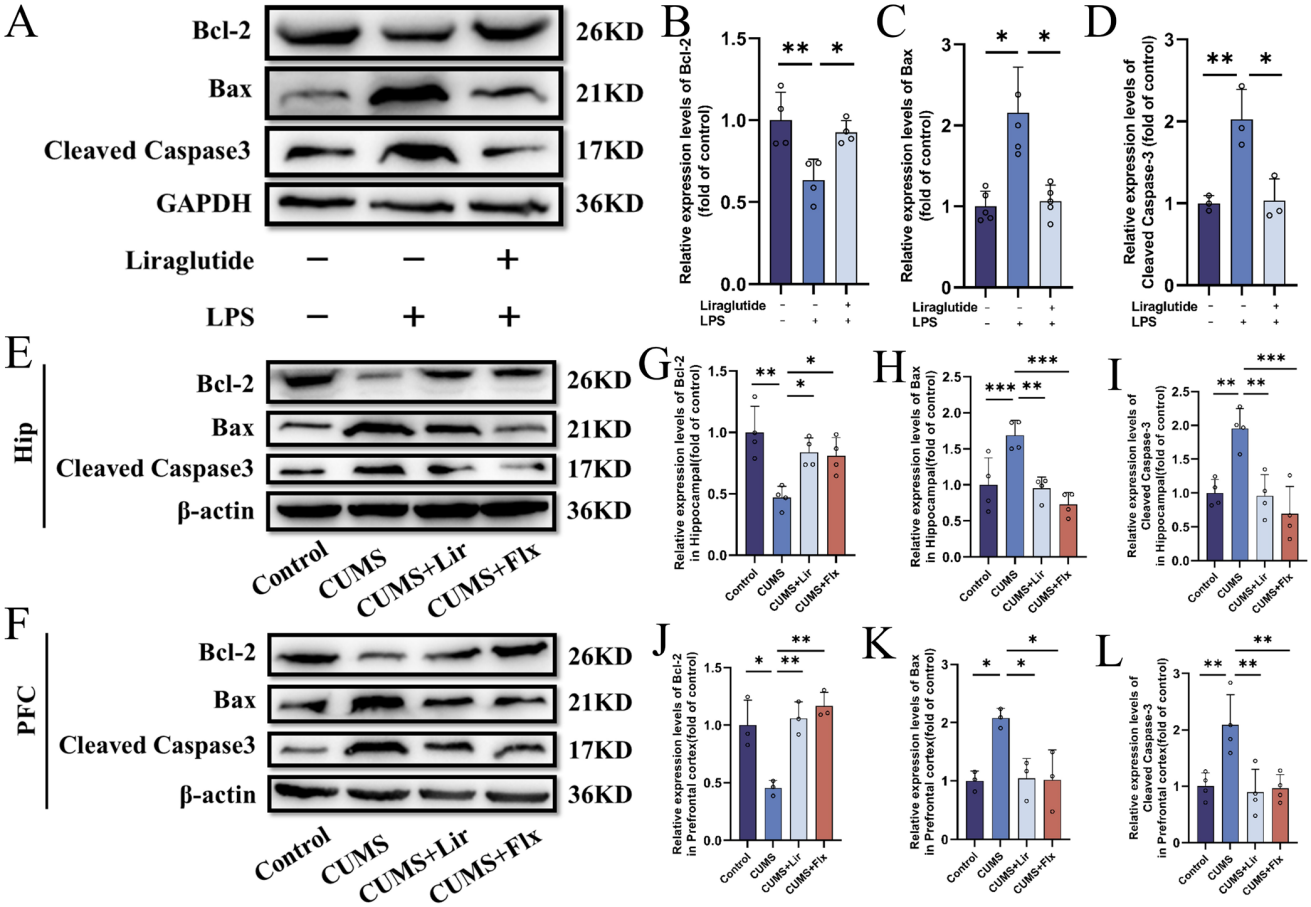
Neuroprotective effects of Liraglutide in the treatment of depression. (A) Representative WB images from in vitro experiments. Quantitative analysis of Bcl‐2 (B), Bax (C) and Cleaved Caspase‐3 (D) protein levels in BV2 cells. (E, F) Representative WB images from in vivo experiments. Quantitative analysis of Bcl‐2 (G, J), Bax (H, K) and Cleaved Caspase‐3 (I, L) protein levels in the HIP and PFC tissues of each group of mice (*n* = 3–5). Normally distributed data were analysed by one‐way ANOVA with Bonferroni or Tamhane's T2 post hoc tests, whereas non‐normally distributed data were assessed using the Kruskal–Wallis test. Data are presented as mean ± SEM. *p < 0.05, **p < 0.01, ***p < 0.001.

We collected the supernatants from BV2 cells in the Control group, LPS group and LPS + Lir group and added them to neuronal HT22 cells for 24 h to establish an ‘indirect co‐culture system of microglia and neurons’. Then we collected HT22 cells from each group and analysed apoptosis‐related proteins using WB to observe the effects of BV2 microglial supernatants on HT22 neurons. As shown in Figure 11A–D, compared to the LPS group, HT22 cells cultured with LPS + Lir‐stimulated BV2 supernatant showed increased Bcl‐2 protein levels (*p =* 0.035) and decreased Bax (*p =* 0.028) and Cleaved Caspase‐3 (*p =* 0.012) levels. We conducted further verification through animal experiments (Figure 11E–L) and obtained the same results.

In conclusion, Lir enhances the anti‐apoptotic capability of HT22 neuronal cells co‐cultured with LPS‐stimulated BV2 cells, demonstrating neuroprotective effects.

## 4 Discussion

Although clinical treatments for depression have achieved some success, limitations such as single‐target therapies, notable side effects and drug resistance persist remain [31, 32]. Therefore, alternative supplementary therapies need to be explored.

Previous studies have indicated that GLP‐1RAs can alleviate multi‐system diseases, such as retinal, renal and hepatic diseases by inhibiting oxidative stress [33, 34, 35]. Despite these findings, few experiments have explored the antioxidative stress effects of Lir in depression. In this experiment, we used network pharmacology to identify common targets of ‘Lir in depression, neuroinflammation and microglia’. Network analysis revealed key targets including inflammatory factors, apoptosis‐related proteins and HMGB1, Nrf2, among others. Furthermore, we found that Lir inhibits oxidative stress formation. In cellular experiments, Lir suppressed ROS production induced by LPS in BV2 cells, balanced the protein levels of Bcl‐2 and Bax in HT22 cells within the ‘microglia‐neuron co‐culture system,’ and inhibited apoptosis‐related protein Cleaved Caspase‐3. In both CUMS‐exposed mice and LPS‐induced BV2 depression models, we found that Lir treatment significantly reduced MDA levels, increased GSH content and enhanced SOD activity, highlighting its role in regulating redox balance and protecting cells from damage. Excess ROS can lead to neuronal membrane lipid peroxidation, protein dysfunction and genetic mutations, thereby impairing normal neuronal function and causing neuronal damage and apoptosis in brain regions [36] involved in regulating mood, cognition and memory, such as the HIP and PFC areas, which are relevant to depression formation [37, 38]. Previous studies have demonstrated that GLP‐1RAs have neuroprotective effects [39, 40]. Our experiment shows that the Lir improves depressive‐like behaviour in mice. H&E and Nissl staining indicate that Lir prevents neuronal cell damage in CUMS‐induced depressed mice. Apoptosis‐related protein detection in HIP, PFC tissues and HT22 cells indicates Lir has anti‐apoptotic effects. These findings suggest that Lir may be used as a potential antidepressant by alleviating excessive oxidative stress damage in depression, inhibiting neuronal damage, and thereby improving depressive‐like behaviour in mice.

Previous studies have found reduced expression of Nrf2 pathway‐related proteins in the cerebral cortex of patients with major depressive disorder, and *Nrf2* knockout mice exhibit depressive‐like behaviours [41, 42], suggesting that Nrf2 may play a critical role in the pathophysiology of depression. In this study, it was demonstrated that Lir treatment increased the expression of total Nrf2 in CUMS‐depressed mice. Further analysis of Nrf2 levels in the nucleus and cytoplasm revealed that Lir exerts its antioxidative stress effects by promoting Nrf2 nuclear translocation, alleviating depressive‐like behaviours in mice. This finding was also confirmed in cellular experiments.

Microglia, the primary immune cells in the central nervous system, become activated in depression and release inflammatory factors and neurotoxic substances, leading to neuroinflammation and damage [2, 43]. Previous studies have found that Lir can alleviate central nervous system demyelinating diseases [44], neuroinflammatory diseases [45] and diabetic neuropathic pain by inhibiting microglial activation [46]. In our in vivo experiments, Lir was proven to reduce the expression of inflammatory factors in the HIP and PFC of depressed mice. Furthermore, in vitro experiments demonstrated that Lir decreased the mRNA levels of inflammatory substances IL‐1β, IL‐6 and TNF‐α in LPS‐stimulated BV2 cells. These results suggest that Lir exerts antidepressant effects by inhibiting neuroinflammation, which can be weakened by the Nrf2 inhibitor ML385. This indicates that the anti‐neuroinflammatory action of Lir may be achieved through Nrf2 activation.

In depression, there is a close connection between inflammation and oxidative stress [47, 48]. Oxidative stress causes cellular damage, releasing damage‐associated molecules that can trigger inflammatory responses [49]. ROS can damage cell membranes and organelles, directly activating pro‐inflammatory signalling pathways and increasing the expression and release of inflammatory factors [50]. Damaged neurons and activated microglia cause HMGB1 to transfer from the nucleus to the cytoplasm and eventually release it extracellularly, interacting with TLRs to participate in the inflammatory response [51]. Previous studies have shown that activating Nrf2 can decrease HMGB1, inhibiting oxidative stress damage. Au inhibits the HMGB1‐TLR4 signalling pathway‐mediated neuroinflammation process, which depends on Nrf2 expression [52]. In both in vitro and in vivo experiments, activation of Nrf2 can suppress the inflammatory response by inhibiting HMGB1 release [53]. Tim‐3 increases HMGB1 levels by inhibiting Nrf2 expression, leading to inflammation following subarachnoid haemorrhage [54]. Our research indicates that Lir inhibits the production of inflammatory factors mediated by the HMGB1‐TLR4 signalling pathway, thus exerting antidepressant effects. However, this effect was reversed by the Nrf2 inhibitor ML385, suggesting that the inhibition of the HMGB1‐TLR4 signalling pathway may depend on Nrf2 expression. Next, to explore the potential mechanism by which Lir activates Nrf2, we examined the activity of the PI3K/AKT pathway in BV2 cells. Our findings further confirmed that Lir can activate Nrf2 via the PI3K/AKT pathway.

Our study combined network pharmacology with in vivo and in vitro experiments to verify that Nrf2 and HMGB1 are important targets for Lir in the treatment of depression. This may provide a new strategy for depression therapy.

The HIP is closely associated with emotional regulation, stress response and neuroplasticity, and is considered one of the classical target regions affected by stress [55, 56]. Many studies have shown that patients with depression often exhibit reduced hippocampal volume and neuronal damage. Moreover, the neuroprotective effects of antidepressants on the hippocampus are positively correlated with their therapeutic outcomes [57, 58, 59]. The PFC plays a key regulatory role in cognition, decision‐making and emotional control. Its dysfunction is often linked to negative emotions, behavioural withdrawal and reduced stress resilience [60]. Therefore, from a mechanistic perspective, focusing on these two regions allows for a more targeted investigation into the core therapeutic effects of Lir in depression. It is worth noting that GLP‐1R is widely distributed in the central nervous system. In addition to the HIP and PFC, it is also strongly expressed in brain regions closely related to emotional regulation, such as the nucleus tractus solitarius (NTS) of the brainstem, the amygdala and the hypothalamus [60]. Neurons in the NTS can project to the nucleus accumbens (NAc), which plays a key role in the regulation of reward pathways and is closely linked to anhedonia [61]. The amygdala has been widely studied for its involvement in emotional processing and anxiety responses [62], whereas the hypothalamus is closely related to stress responses and the regulation of physiological functions [63].

Therefore, future studies could explore whether Lir exerts its antidepressant effects by modulating inflammation and oxidative stress in these regions, potentially acting through broader neural circuits.

## 5 Conclusions

In conclusion, our study is the first to demonstrate that Lir exerts antidepressant effects through the PI3K/Nrf2/HMGB1 signalling pathway. The findings of this paper may provide new strategies for the treatment of depression.

## Author Contributions

**Jiangjin Sun:** conceptualization (lead), data curation (lead), formal analysis (lead), investigation (lead), validation (lead), writing – original draft (lead). **Xiying Fu:** project administration (equal), resources (equal), visualization (equal). **Yaqi Liu:** investigation (equal), methodology (equal), software (equal). **Tian Wang:** conceptualization (equal), data curation (equal), formal analysis (equal). **Xing Zhao:** investigation (equal), methodology (equal), resources (equal). **Ranji Cui:** funding acquisition (equal), project administration (equal), writing – review and editing (equal). **Wei Yang:** conceptualization (equal), data curation (equal), formal analysis (equal), funding acquisition (equal), methodology (equal), project administration (equal), writing – review and editing (lead).

## Conflicts of Interest

The authors declare no conflicts of interest.

## Supporting information


Data S1

## Data Availability

Data will be made available on request.
