# Supplementary material for: Exploring the Effect and Mechanism of Liraglutide in Treating Depression Based on Network Pharmacology and Experimental Analysis
Source: J Cell Mol Med. 2025 Jun 3;29(11):e70630. doi: 10.1111/jcmm.70630 (PMC12133450; doi:10.1111/jcmm.70630)
Supplement: Supplementary file 1 — Data S1 [file JCMM-29-e70630-s001.docx]

**Supplementary materials**

**Exploring the effect and mechanism of Liraglutide in treating depression based on network pharmacology and experimental analysis**

Jiangjin Sun ^a, b^, Xiying Fu ^a, c^, Yaqi Liu ^a,b^, Tian Wang ^a, b^, Xing Zhao ^b^, Ranji Cui ^a,*^, Wei Yang ^a, b,*^

^a^ *Jilin Provincial Key Laboratory on Molecular and Chemical Genetic, The Second Clinical Medical College of Jilin University, Changchun, China*

^b^ *Department of Neurology, The Second Clinical Medical College of Jilin University, Changchun, China*

^c^ *Department of Endocrinology, The Second Clinical Medical College of Jilin University, Changchun, China.*

** Corresponding authors*

*E-mail: cuiranji@jlu.edu.cn (R. Cui), wyang2002@jlu.edu.cn (W. Yang)*

# Supplementary methods

# 2. Materials and methods

## 2.1. Network pharmacology

### 2.2.1 Screening and prediction of drug component targets and depression-related targets

The chemical structure of the Lir was searched from the PubChem open chemistry database (https://pubchem.ncbi.nlm.nih.gov) of the National Institutes of Health (NIH)[1]. Target information was then obtained using the SEA database (https://sea.bkslab.org/)[2], Swiss Target Prediction (http://www.swisstargetprediction.ch/)[3], and TargetNet (http://targetnet.scbdd.com/home/index/)[4]. Standard gene names were converted using the Universal Protein Resource (https://www.uniprot.org/)[5]. Relevant genes were selected from databases such as GeneCards (https://www.genecards.org)[6], OMIM (https://www.omim.org)[7], and CTD (https://ctdbase.org)[8] using keywords like "depression," "microglia," "inflammation," and "neuroinflammation." Duplicate data were consolidated and removed.

### 2.1.2. Identification of potential targets

The obtained drug and disease targets were imported into the Venny diagram (https://www.bioinformatics.com.cn/static/others/jvenn/example.html)[9] to identify their intersection. The overlapping targets were identified as potential targets for Lir in the intervention of neuroinflammation-related depression.

### 2.1.3. Construction of Protein-Protein Interaction (PPI) network and core target screening

The identified potential targets were imported into the STRING database (https://string-db.org/) [10]to construct a PPI network model. The resulting data were then imported into Cytoscape 3.9.1 software to visualize and analyze the PPI network[11].

### 2.1.4. Gene ontology (GO) functional enrichment analysis and Kyoto Encyclopedia of genes and genomes (KEGG) pathway enrichment analysis

The intersecting genes were imported into the DAVID database (https://david.ncifcrf.gov/) for GO biological function enrichment analysis and KEGG pathway enrichment analysis [12]. Bar and bubble charts were generated using the Bioinformatics platform (https://www.bioinformatics.com.cn/).

## 2.2. Experimental animals and drugs

Male C57BL/6 mice (aged 8-10 weeks, weighing 18-23 g) were purchased from Liaoning Changsheng Life Sciences Ltd (Benxi, Liaoning, China). The experimental animals were housed according to ethical principles for laboratory animal care (12-hour light-dark cycle, temperature 22±2°C), with enough food and water. All procedures were conducted according to the standards of the Laboratory Animal Guideline for Ethical Review of Animal Welfare (GB/T 35892-2018) and were approved by the Ethics Committee of Jilin University (approval No.2020/150; December 9, 2020). Lir (Novo Nordisk, Denmark) was dissolved in physiological saline. Fluoxetine (Flx, PHR1394, Sigma-Aldrich, USA) was dissolved in physiological saline. The Nrf2 inhibitor ML385 (HY-100523, Med Chem Express, China) was prepared as a stock solution in DMSO. The HMGB1 inhibitor Glycyrrhizin (GL, 50531, Sigma-Aldrich, USA) was prepared as a stock solution in DMSO. The PI3K pathway inhibitor LY294002 (HY-10108, Med Chem Express, China) was prepared as a stock solution in DMSO. All drugs were diluted to the correct concentrations before use.

## 2.3. Experimental design

### 2.3.1. Animal experiments

#### Experiment groups and drug dose

In the first experiment, mice were randomly divided into 6 groups (n = 10 per group): Control, CUMS, CUMS + low-dose Lir [50 μg/kg/day[13], intraperitoneally (i.p.)], CUMS + medium-dose Lir (150 μg/kg/day[14], i.p.), CUMS + high-dose Lir (300 μg/kg/day[15], i.p.), and CUMS + fluoxetine (20 mg/kg/day[16], i.p.). The experimental timeline is shown in **Fig. S1**. After 4 weeks of CUMS stimulation, mice received intraperitoneal injections for 2 weeks, while the Control and CUMS model groups received saline. Behavioral testing and tissue collection were subsequently performed.

In the second experiment, mice were randomly divided into 4 groups (n = 13 per group): Control, CUMS, CUMS + Lir, and CUMS + Lir + ML385. Mice in the inhibitor group received an intraperitoneal injection of ML385 (30 mg/kg/day[17]) 30 min before the Lir injection, while the other groups received saline injections. Behavioral testing and tissue collection were performed after 2 weeks.

In the third experiment, mice were randomly divided into 4 groups (n = 10 per group): Control, CUMS, CUMS + Lir, and CUMS + GL (20 mg/kg/day[18], i.p.). The Control and CUMS model groups received saline injections. After 2 weeks of treatment, behavioral testing, and tissue collection were performed.

In the fourth experiment, mice were divided into four groups: Control, CUMS, CUMS + Lir, and CUMS + Lir + LY294002 (25 mg/kg/day[19, 20], i.p.). In the inhibitor group, LY294002 was administered by intraperitoneal injection 1 h before Lir treatment.

#### Behavioral tests

#### Open Field Test (OFT)

Mice were placed in the center of a square acrylic apparatus (50 cm × 50 cm × 30 cm) and a camera recorded their movements for 6 min[21]. After each test, the apparatus was cleaned of mouse droppings and sprayed with alcohol to prevent the smell of the previous mouse from affecting subsequent tests. The results were analyzed using an animal movement tracking system (Noldus, EthoVision XT 13). The number of entries into the central area was used to indicate anxiety-like behavior. The total distance traveled and the movement speed in the open field test were used to indicate spontaneous movement activity.

#### Tail Suspension Test (TST)

A piece of tape was attached 2 cm from the tip of the mouse's tail, and the tape was attached to a suspension device, positioning the mouse approximately 20 cm above the ground in an inverted posture. The mouse's behavior was recorded with a camera for 6 min. The immobility time during the last 4 min was noted. Immobility time was defined as the period during which the mouse ceased struggling, remained suspended vertically, and stayed motionless. The detailed process can be found in our previous study[22].

#### Forced Swim Test (FST)

Mice were individually placed in a transparent glass cylinder filled with water (25±1°C) (height 25 cm, diameter 11 cm, water depth 12 cm) for 6 min, and their swimming behavior was recorded with a camera. After each test, the water was changed to prevent the smell of the previous mouse from affecting subsequent tests. The immobility time during the last 4 min was counted. Immobility time was defined as the period during which the mouse remained motionless, excluding the minimal movements necessary to keep its head above water. Swimming time was defined as the period during which the mouse was actively swimming, with its body parallel to the bottom of the cylinder. The detailed process can be found in our previous study[22].

### 2.3.2. In Vitro experiments

#### Method of cell viability assay

Cell viability was assessed using a CCK8 kit (Pumoke, Wuhan, China). BV2 cells were subjected to intervention when they reached approximately 70% cell density in 100 mm culture dishes. Cells were grouped using a 96-well plate, 100 μL of medium was added to each well, and 8000 cells were inoculated. Cells were pretreated with 0 nM, 2 nM, 10 nM, 100 nM, and 500 nM of Lir for 1 h, respectively, followed by incubation with 1 μg/ml of LPS (L2630, Sigma-Aldrich, USA) for 24 h[23]. Cells were divided into the LPS group and 4 drug intervention groups according to Lir concentration, and the control group used a normal medium, wells without cells were used as blank groups. After culturing, 10 μl of CCK-8 solution was added to each well and incubated for 1-2 h. Absorbance at 450 nm was measured using a microplate reader (Thermo Scientific, USA). Cell viability was calculated using the equation: Viability = [A(experimental) - A(blank)] / [A(control) - A(blank)] × 100%.

#### Process of cell culture

BV2 microglial cell line (Pricella, Wuhan, China) and mouse hippocampus HT22 cell line (Microread Genetics, Beijing, China) were cultured in DMEM medium (Gibco, USA) supplemented with 10% fetal bovine serum (Cell-Box, Hong Kong). The cells were cultured in a constant-temperature incubator at 37°C with 5% CO_2_. The experimental timeline is shown in **Fig. S2**. In the in vitro experiment, when the BV2 cell density reached approximately 70%, drug treatment was applied. First, cells were pretreated with 5 nM Nrf2 inhibitor ML385 for 2 h[24], followed by 100 nM Lir treatment for 1 h. Afterward, cells were stimulated with 1 μg/ml LPS for 24 h, and then collected for subsequent experiments (the drug concentrations of Lir[25]and LPS[26] were mainly based on previous studies and published articles). Additionally, for the PI3K/Akt pathway inhibition assay, BV2 cells were pretreated with 10 μM LY294002 (a PI3K inhibitor) for 1 h before Lir treatment[27].

#### Indirect Co-Culture of BV2 and HT22 cells

The culture media from the BV2 cell groups (control, LPS, LPS + Lir) in 100 mm dishes were collected and centrifuged at 1000 rpm for 5 min. And the supernatants were then added to HT22 cells that had reached 70% cell density. After 24 h of incubation, the HT22 cells were collected for further experiments.

### 2.3.3. Western Blot (WB)

Hippocampal (Hip) and prefrontal cortex (PFC) tissue or cells were lysed in RIPA buffer containing 1% PMSF, then the samples were sonicated and centrifuged at 12,000 rpm for 20 min at 4°C. The supernatant was collected and mixed with loading buffer, and the mixture was denatured by heating at 95°C for 10 min. Nuclear and cytoplasmic proteins were extracted using a commercial kit (DE201, TransGen Biotech, China). Protein samples were separated on 10% SDS-polyacrylamide gels and transferred onto polyvinylidene fluoride (PVDF) membranes (IPVH00010, Millipore). The membranes were blocked with 5% non-fat milk for 1 h and then incubated overnight at 4°C with the following primary antibodies: rabbit anti-Nrf2 (1:5000, 16396-1-AP, Proteintech), rabbit anti-HMGB1 (1:1000, 10829-1-AP, Proteintech), mouse anti-Keap1 (1:1000, 60027-1-Ig, Proteintech), rabbit anti-HO-1 (1:1000, 10701-1-AP, Proteintech), rabbit anti-NQO1 (1:2000, 11451-1-AP, Proteintech), mouse anti-TLR4 (1:1000, 66350-1-Ig, Proteintech), rabbit anti-PI3K (1:1000, #4292, Cell Signaling), rabbit anti-p-PI3K (1:1000, #4228, Cell Signaling), rabbit anti-AKT (1:500, WL0003b, Wanlei Bio), rabbit anti-p-AKT (1:500, WL0003a, Wanlei Bio), mouse anti-Bcl-2 (1:2000, 68103-1-Ig, Proteintech), rabbit anti-Bax (1:2000, 50599-2-Ig, Proteintech), rabbit anti-Cleaved Caspase3 (1:1000, 9664, Cell Signaling), rabbit anti-Lamin B1 (1:5000, 12987-1-AP, Proteintech), mouse anti-β-actin (1:2000, TA-09, Zhong Shan-Golden Bridge), and mouse anti-GAPDH (1:5000, 60004-1-Ig, Proteintech). The membranes were then washed three times with TBST for 5 min each and incubated with corresponding secondary antibodies (1:5000, ZB-2305/ZB-2301, Zhong Shan-Golden Bridge) at room temperature for 1 h. Protein bands were detected using enhanced chemiluminescence (ECL, PK10002, Proteintech). The optical density of the protein bands was analyzed using ImageJ software.

### 2.3.4. Process for paraffin embedding and sectioning of brain tissue

The process of brain tissue paraffin embedding and sectioning is as follows. First, mice were deeply anesthetized and perfused with 40 ml of physiological saline, followed by 40 ml of 4% paraformaldehyde (PFA). Then the mouse brains were fixed in 4% PFA for 24 h, and immersed in 75% ethanol for 4 h, 85% ethanol for 2 h, 90% ethanol for 2 h, 95% ethanol for 1 h, absolute ethanol I for 30 min, absolute ethanol II for 30 min, alcohol-benzene solution for 10 min, xylene I for 10 min, and xylene II for 10 min respectively, achieved tissues dehydrated and rendered transparent. Finally, tissues were embedded in paraffin and sectioned to a thickness of 4 μm using a microtome[28].

### 2.3.5. Hematoxylin and Eosin (H&E) staining and Nissl staining

Brain slices were baked, deparaffinized, and rehydrated, then stained with H&E and Nissl, and finally sealed with neutral resin. Images were observed and captured using an optical microscope (Olympus, Japan), and the number of damaged neurons and Nissl bodies were quantified using ImageJ software (version 1.8. 0, NIH, Bethesda, USA).

### 2.3.6. Immunofluorescent staining

After behavioral tests, the mice were anesthetized for perfusion. Then, the entire brain was carefully removed and immersed in 4% PFA, fixed at room temperature overnight. The brain was dehydrated using a sucrose gradient of 10%, 20%, and 30%, embedded, and stored at -80°C for use. The frozen mouse brain was then placed in a frozen slicer to cut 20 μm brain tissue sections for immunofluorescence staining. During staining, the tissue sections were washed with PBS, treated with sodium citrate solution for antigen restoration, and then blocked with 5% goat serum for 1 h. The sections were incubated overnight at 4°C with primary antibodies [Iba-1 (1:500, 019-19741, Wako) and GLP-1R (1:50, sc-390774, Santa Cruz)]. The next day, the sections were incubated at room temperature for 1 h with secondary antibodies [CY3- goat anti-rabbit IgG (H+L) (1:100, PMK-014096S, Bioprimacy) and FITC- goat anti-mouse IgG (H+L) (1:100, PMK-014093S, Bioprimacy)]. The sections were then incubated with DAPI solution for 15 min at room temperature in the dark. Afterward, coverslips were applied under light-protected conditions, and the sections were mounted with glycerol. The samples were observed and photographed using a fluorescence microscope.

### 2.3.7. Method of Enzyme-Linked Immunosorbent Assay (ELISA)

The levels of Interleukin-1β (IL-1β), Interleukin-6 (IL-6), Tumor Necrosis Factor-α (TNF-α), and peripheral serum corticosterone in mouse Hip and PFC tissues were measured using an ELISA kit (Jianglai Biological, Shanghai, China). Tissue samples were weighed and homogenized with PBS at a ratio of 1 g tissue to 9 ml PBS on ice. The homogenate was then centrifuged at 5000 rpm for 15 min at 4°C, and the supernatant was collected for analysis. According to the kit instructions, 100 μl of either standard or sample dilution was added to each well and incubated at 37°C for 1 h. Subsequently, 100 μl of biotinylated antibody working solution was added to each well, covered with a sealing film, and incubated at 37°C for 60 min. After washing, 100 μl of enzyme conjugate working solution was added to each well and incubated at 37°C for 30 min. The wells were then washed again, and 90 μl of substrate solution was added, covered with sealing film, and incubated at 37°C in the dark for 15 min. Finally, 50 μl of stop solution was added to each well, and the optical density (OD) values were immediately measured at a wavelength of 450 nm. A standard curve was constructed using the OD values of the standards to calculate the concentrations of inflammatory factors in the tissue samples.

### 2.3.8. Detection of oxidative stress factor levels

After weighing the tissues, they were homogenized in physiological saline at a ratio of 1:9 (weight in grams: volume in milliliters) and centrifuged at 5000 rpm for 10 min, and the supernatant was collected for analysis. Cells were similarly homogenized in physiological saline, and the supernatant was obtained. Protein concentration was determined using a BCA Protein Assay Kit (P0010S, Beyotime, China). Subsequently, oxidative stress indicators, including Malondialdehyde (MDA, S0131S, Beyotime, China), Glutathione (GSH, A006-2-1, Nanjing Jiancheng, China), and Superoxide Dismutase (SOD, A001-3, Nanjing Jiancheng, China), were measured by adding standards, samples, and working solutions according to the kit instructions. OD of each well was measured using a microplate reader, and a standard curve based on OD values of the standards was used to calculate oxidative stress marker levels.

### 2.3.9. Method of Quantitative Real-Time PCR (qRT-PCR)

Total RNA was extracted from tissues or cells by sequentially adding TRIzol (TransGen Biotech, China), chloroform, isopropanol, 75% ethanol, etc. RNA concentration and purity were determined using a NanoDrop One spectrophotometer. Total RNA was reverse-transcribed into cDNA using a kit (AU341, TransGen Biotech, China). The expression levels of mRNA in tissues were then detected using SYBR Green qPCR Master Mix (AQ601, TransGen Biotech, China) on a real-time PCR system (CFX, Bio-Rad, USA). All samples were run in triplicate, and mRNA expression levels were normalized to β-actin mRNA. Quantitative analysis of mRNA expression levels was performed using the 2^−ΔΔCT^ method. The primer sequences are listed in **Table S1**.

### 2.3.10. Tyramide Signal Amplification (TSA) for immunofluorescence double staining

After drug treatment of BV2 cells, the cells were collected by centrifugation and washed twice with PBS. 4% PFA was fixed at room temperature for 15 min, and the supernatant was removed by centrifugation after three washes with PBS. Then the cell concentration was adjusted and 30ul of cell dilution was dripped onto the slides, which were thoroughly dried in an oven. The slide was placed in an antigen retrieval box at 95°C for 10 min for antigen retrieval. Endogenous peroxidase was inactivated with 3% H_2_O_2_, followed by permeabilization with 0.3% Triton-X 100 for 10 min at room temperature, and blocking with 10% goat serum for 30 min. The cells were then incubated overnight at 4°C with rabbit anti-Nrf2 antibody (1:50). The next day, after washing off the primary antibody, the slide was incubated with the corresponding secondary antibody for 1 h, then washed, and the signal was amplified using TSA dye CY3-Tyramide, followed by termination with PBS. The slide was again subjected to antigen retrieval at 95°C to remove the bound primary and secondary antibodies. After washing three times with PBS, the cells were incubated overnight at 4°C with rabbit anti-HMGB1 antibody (1:50). On the third day, after incubating with the corresponding secondary antibody for 1 h and washing the slide, the signal was amplified using TSA dye FITC-Tyramide. Finally, the slide was stained with DAPI, imaging was performed using an inverted fluorescence microscope (Olympus, Japan), and fluorescence intensity was analyzed using ImageJ software.

### 2.3.11. Detection of Reactive Oxygen Species (ROS) levels

BV2 cells were cultured in six-well plates, and ROS levels were detected using a ROS kit ((Pumoke, Wuhan China) at the end of drug intervention. H2DCFH-DA was diluted in serum-free culture medium at a ratio of 1:1000 to achieve a final concentration of 10 μM. After removing the culture medium, cells were washed three times with PBS to remove surface debris. Subsequently, 1 ml of the diluted H2DCFH-DA solution was added to each well, and cells were then incubated at 37°C in a cell culture incubator for 30 min to load the probe. After the incubation, cells were washed three times with serum-free culture medium to remove excess H2DCFH-DA that did not enter the cells. Immediately after that, cells were observed and images were captured using an inverted fluorescence microscope. ImageJ software was employed for fluorescence intensity analysis.

# References

[1] S. Kim, J. Chen, T. Cheng, et al. 2021. PubChem in 2021: new data content and improved web interfaces. *Nucleic Acids Res.* 49(D1) D1388-d1395. <http://dx.doi.org/10.1093/nar/gkaa971>.

[2] M.J. Keiser, B.L. Roth, B.N. Armbruster, et al. 2007. Relating protein pharmacology by ligand chemistry. *Nat. Biotechnol.* 25(2) 197-206. <http://dx.doi.org/10.1038/nbt1284>.

[3] A. Daina, O. Michielin, V. Zoete. 2019. SwissTargetPrediction: updated data and new features for efficient prediction of protein targets of small molecules. *Nucleic Acids Res.* 47(W1) W357-w364. <http://dx.doi.org/10.1093/nar/gkz382>.

[4] Z.J. Yao, J. Dong, Y.J. Che, et al. 2016. TargetNet: a web service for predicting potential drug-target interaction profiling via multi-target SAR models. *J. Comput. Aided Mol. Des.* 30(5) 413-424. <http://dx.doi.org/10.1007/s10822-016-9915-2>.

[5] 2021. UniProt: the universal protein knowledgebase in 2021. *Nucleic Acids Res.* 49(D1) D480-d489. <http://dx.doi.org/10.1093/nar/gkaa1100>.

[6] G. Stelzer, N. Rosen, I. Plaschkes, et al. 2016. The GeneCards Suite: From Gene Data Mining to Disease Genome Sequence Analyses. *Curr. Protoc. Bioinformatics*. 54 1.30.31-31.30.33. <http://dx.doi.org/10.1002/cpbi.5>.

[7] J.S. Amberger, C.A. Bocchini, A.F. Scott, et al. 2019. OMIM.org: leveraging knowledge across phenotype-gene relationships. *Nucleic Acids Res.* 47(D1) D1038-d1043. <http://dx.doi.org/10.1093/nar/gky1151>.

[8] A.P. Davis, T.C. Wiegers, R.J. Johnson, et al. 2023. Comparative Toxicogenomics Database (CTD): update 2023. *Nucleic Acids Res.* 51(D1) D1257-d1262. <http://dx.doi.org/10.1093/nar/gkac833>.

[9] P. Bardou, J. Mariette, F. Escudié, et al. 2014. jvenn: an interactive Venn diagram viewer. *BMC Bioinformatics*. 15(1) 293. <http://dx.doi.org/10.1186/1471-2105-15-293>.

[10] D. Szklarczyk, R. Kirsch, M. Koutrouli, et al. 2023. The STRING database in 2023: protein-protein association networks and functional enrichment analyses for any sequenced genome of interest. *Nucleic Acids Res.* 51(D1) D638-d646. <http://dx.doi.org/10.1093/nar/gkac1000>.

[11] P. Shannon, A. Markiel, O. Ozier, et al. 2003. Cytoscape: a software environment for integrated models of biomolecular interaction networks. *Genome Res.* 13(11) 2498-2504. <http://dx.doi.org/10.1101/gr.1239303>.

[12] B.T. Sherman, M. Hao, J. Qiu, et al. 2022. DAVID: a web server for functional enrichment analysis and functional annotation of gene lists (2021 update). *Nucleic Acids Res.* 50(W1) W216-w221. <http://dx.doi.org/10.1093/nar/gkac194>.

[13] M. Prá, G.K. Ferreira, A.H. de Mello, et al. 2016. Single dose and repeated administrations of liraglutide alter energy metabolism in the brains of young and adult rats. *Biochem. Cell Biol.* 94(5) 451-458. <http://dx.doi.org/10.1139/bcb-2016-0016>.

[14] P. Koshal, P. Kumar. 2016. Effect of Liraglutide on Corneal Kindling Epilepsy Induced Depression and Cognitive Impairment in Mice. *Neurochem. Res.* 41(7) 1741-1750.

<http://dx.doi.org/10.1007/s11064-016-1890-4>.

[15] M.K. Seo, S. Jeong, D.H. Seog, et al. 2023. Effects of liraglutide on depressive behavior in a mouse depression model and cognition in the probe trial of Morris water maze test. *J. Affect. Disord.* 324 8-15. <http://dx.doi.org/10.1016/j.jad.2022.12.089>.

[16] W. Jiang, Q. Chen, P. Li, et al. 2017. Magnesium Isoglycyrrhizinate attenuates lipopolysaccharide-induced depressive-like behavior in mice. *Biomed. Pharmacother.* 86 177-184. <http://dx.doi.org/10.1016/j.biopha.2016.12.033>.

[17] R. Dang, M. Wang, X. Li, et al. 2022. Edaravone ameliorates depressive and anxiety-like behaviors via Sirt1/Nrf2/HO-1/Gpx4 pathway. *J. Neuroinflammation*. 19(1) 41. <http://dx.doi.org/10.1186/s12974-022-02400-6>.

[18] T.Y. Wu, L. Liu, W. Zhang, et al. 2015. High-mobility group box-1 was released actively and involved in LPS induced depressive-like behavior. *J. Psychiatr. Res.* 64 99-106. <http://dx.doi.org/10.1016/j.jpsychires.2015.02.016>.

[19] X. Xing, J. Zhang, K. Wu, et al. 2019. Suppression of Akt-mTOR pathway rescued the social behavior in Cntnap2-deficient mice. *Sci. Rep.* 9(1) 3041. <http://dx.doi.org/10.1038/s41598-019-39434-5>.

[20] J.C. Yang, S.C. Wu, C.S. Rau, et al. 2014. Inhibition of the phosphoinositide 3-kinase pathway decreases innate resistance to lipopolysaccharide toxicity in TLR4 deficient mice. *J. Biomed. Sci.* 21(1) 20. <http://dx.doi.org/10.1186/1423-0127-21-20>.

[21] L. Prut, C. Belzung. 2003. The open field as a paradigm to measure the effects of drugs on anxiety-like behaviors: a review. *Eur. J. Pharmacol.* 463(1-3) 3-33.

http://dx.doi.org/10.1016/s0014-2999(03)01272-x.

[22] Z. Cheng, F. Zhao, J. Piao, et al. 2024. Rasd2 regulates depression-like behaviors via DRD2 neurons in the prelimbic cortex afferent to nucleus accumbens core circuit. *Mol. Psychiatry*. <http://dx.doi.org/10.1038/s41380-024-02684-5>.

[23] L.T. Guo, S.Q. Wang, J. Su, et al. 2019. Baicalin ameliorates neuroinflammation-induced depressive-like behavior through inhibition of toll-like receptor 4 expression via the PI3K/AKT/FoxO1 pathway. *J. Neuroinflammation*. 16(1) 95.

http://dx.doi.org/10.1186/s12974-019-1474-8.

[24] J. Che, H. Wang, J. Dong, et al. 2024. Human umbilical cord mesenchymal stem cell-derived exosomes attenuate neuroinflammation and oxidative stress through the NRF2/NF-κB/NLRP3 pathway. *CNS Neurosci. Ther.* 30(3) e14454. <http://dx.doi.org/10.1111/cns.14454>.

[25] F. Jing, Q. Zou, Y. Wang, et al. 2021. Activation of microglial GLP-1R in the trigeminal nucleus caudalis suppresses central sensitization of chronic migraine after recurrent nitroglycerin stimulation. *J. Headache Pain*. 22(1) 86. <http://dx.doi.org/10.1186/s10194-021-01302-x>.

[26] W. Tao, Y. Hu, Z. Chen, et al. 2021. Magnolol attenuates depressive-like behaviors by polarizing microglia towards the M2 phenotype through the regulation of Nrf2/HO-1/NLRP3 signaling pathway. *Phytomedicine*. 91 153692. <http://dx.doi.org/10.1016/j.phymed.2021.153692>.

[27] Y. Xiong, W. Liang, X. Wang, et al. 2024. S100A8 knockdown activates the PI3K/AKT signaling pathway to inhibit microglial autophagy and improve cognitive impairment mediated by chronic sleep deprivation. *Int. Immunopharmacol.* 143(Pt 2) 113375. <http://dx.doi.org/10.1016/j.intimp.2024.113375>.

[28] Y.J. Dong, N.H. Jiang, L.H. Zhan, et al. 2021. Soporific effect of modified Suanzaoren Decoction on mice models of insomnia by regulating Orexin-A and HPA axis homeostasis. *Biomed. Pharmacother.* 143 112141. <http://dx.doi.org/10.1016/j.biopha.2021.112141>.

# Supplementary figures


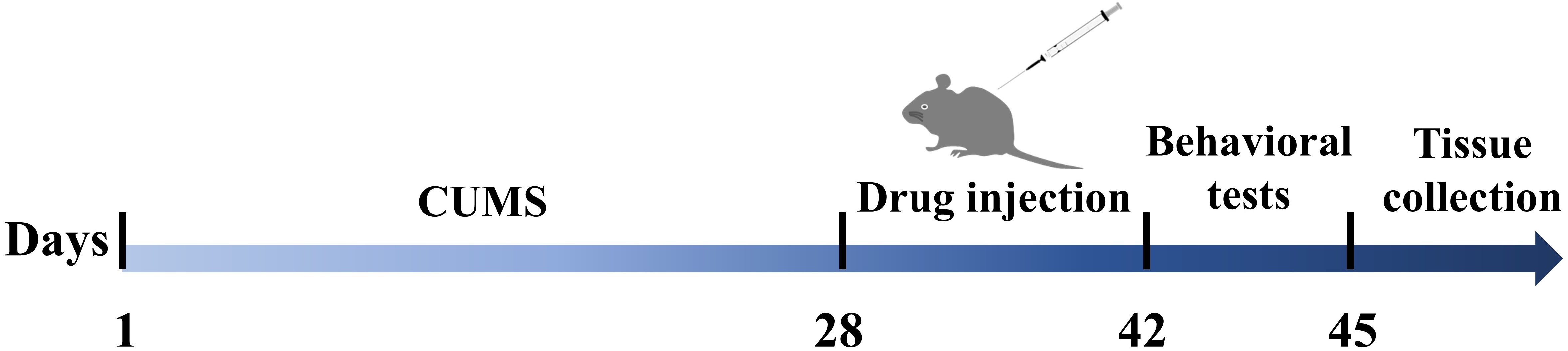


**Fig. S1** Timeline of Animal Experiments.


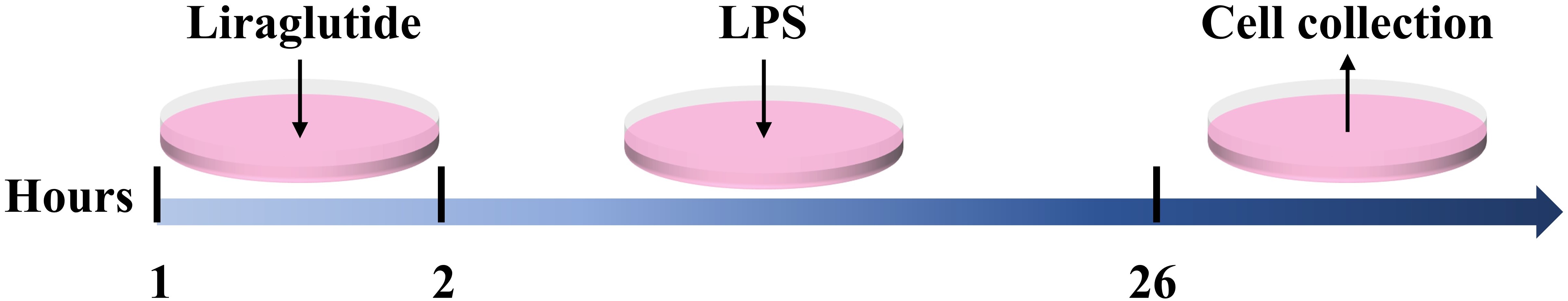


**Fig. S2** Timeline of In Vitro Experiments.





**Fig. S3** Lir suppressed CUMS-induced microglial activation. (A) Representative images of IBa-1 and CD68 staining in the CA1 region of the HIP. (B, C) Quantitative analysis of IBa-1 and CD68 positive areas in the CA1 region. (D) Representative images of IBa-1 and CD68 in the CA3 region. (E, F) Quantitative analysis of IBa-1 and CD68 positive areas in the CA3 region. (G) Representative images of IBa-1 and CD68 in the DG region. (H, I) Quantitative analysis of IBa-1 and CD68 positive areas in the DG region. (J) Representative images of IBa-1 and CD68 in the PFC region. (K, L) Quantitative analysis of IBa-1 and CD68 positive areas in the PFC region. (n = 3), scale bar = 20 μm. Normally distributed data were analyzed by one-way ANOVA with Bonferroni or Tamhane’s T2 post hoc tests, while non-normally distributed data were assessed using the Kruskal-Wallis test. Data are presented as mean ± SEM. **P* < 0.05, ***P* < 0.01, ****P* < 0.001.

# Supplementary tables

**Table S1** The primer sequences of inflammatory factors

| Gene | Primer sequences |
| --- | --- |
| IL-1β | Forward:5’-CTCGCAGCAGCACATCAACAAG-3’  Reverse: 5’-CCACGGGAAAGACACAGGTAGC-3’ |
| IL-6 | Forward:5’-TTCTTGGGACTGATGCTGGTGAC-3’  Reverse: 5’-GTGGTATCCTCTGTGAAGTCTCCTC-3’ |
| TNF-α | Forward:5’-ACGCTCTTCTGTCTACTGAACTTCG-3’ |
|  | Reverse: 5’-TGGTTTGTGAGTGTGAGGGTCTG-3’ |
| β-actin | Forward:5’-GATGGTGGGAATGGGTCAGAAGG-3’ |
|  | Reverse: 5’-TTGTAGAAGGTGTGGTGCCAGATC-3’ |

**Table S2** Target name of genes

| Gene | Target name |
| --- | --- |
| TNF | Tumor necrosis factor |
| AKT1 | AKT serine/threonine kinase 1 |
| IL1B | Interleukin-1 beta |
| EGFR | Epidermal growth factor receptor |
| CASP3 | Caspase 3 |
| BCL2 | BCL2 apoptosis regulator |
| MMP9 | Matrix metallopeptidase 9 |
| SRC | SRC proto-oncogene, non-receptor tyrosine kinase |
| PTGS2 | Prostaglandin-endoperoxide synthase 2 |
| PPARG | Peroxisome proliferator-activated receptor gamma |
| ESR1 | Estrogen receptor 1 |
| HMGB1 | High mobility group box 1 |
| GSK3B | Glycogen synthase kinase 3 beta |
| NFE2L2 | Nuclear factor erythroid 2-related factor 2 |
| MMP2 | Matrix metallopeptidase 2 |
| ERBB2 | Erb-b2 receptor tyrosine kinase 2 |
| CXCR4 | C-X-C motif chemokine receptor |
| VCAM1 | Vascular cell adhesion molecule 1 |
| SIRT1 | NAD-dependent protein deacetylase sirtuin-1 |
| APP | Amyloid beta precursor protein |
